# Glutamate, GABA and Acetylcholine Signaling Components in the Lamina of the *Drosophila* Visual System

**DOI:** 10.1371/journal.pone.0002110

**Published:** 2008-05-07

**Authors:** Agata Kolodziejczyk, Xuejun Sun, Ian A. Meinertzhagen, Dick R. Nässel

**Affiliations:** 1 Department of Zoology, Stockholm University, Stockholm, Sweden; 2 Life Sciences Centre, Dalhousie University, Halifax, Nova Scotia, Canada; Katholieke Universiteit Leuven, Belgium

## Abstract

Synaptic connections of neurons in the *Drosophila* lamina, the most peripheral synaptic region of the visual system, have been comprehensively described. Although the lamina has been used extensively as a model for the development and plasticity of synaptic connections, the neurotransmitters in these circuits are still poorly known. Thus, to unravel possible neurotransmitter circuits in the lamina of *Drosophila* we combined Gal4 driven green fluorescent protein in specific lamina neurons with antisera to γ-aminobutyric acid (GABA), glutamic acid decarboxylase, a GABA_B_ type of receptor, L-glutamate, a vesicular glutamate transporter (vGluT), ionotropic and metabotropic glutamate receptors, choline acetyltransferase and a vesicular acetylcholine transporter. We suggest that acetylcholine may be used as a neurotransmitter in both L4 monopolar neurons and a previously unreported type of wide-field tangential neuron (*Cha*-Tan). GABA is the likely transmitter of centrifugal neurons C2 and C3 and GABA_B_ receptor immunoreactivity is seen on these neurons as well as the *Cha*-Tan neurons. Based on an *rdl*-Gal4 line, the ionotropic GABA_A_ receptor subunit RDL may be expressed by L4 neurons and a type of tangential neuron (*rdl*-Tan). Strong vGluT immunoreactivity was detected in α-processes of amacrine neurons and possibly in the large monopolar neurons L1 and L2. These neurons also express glutamate-like immunoreactivity. However, antisera to ionotropic and metabotropic glutamate receptors did not produce distinct immunosignals in the lamina. In summary, this paper describes novel features of two distinct types of tangential neurons in the *Drosophila* lamina and assigns putative neurotransmitters and some receptors to a few identified neuron types.

## Introduction

One of the most extensively investigated portions of the insect brain is the first synaptic neuropil in the optic lobe of flies, the lamina. This neuropil corresponds in its processing operations to the outer plexiform layer of the vertebrate retina, and indeed since the seminal work of Cajal and Sánchez [1] insect visual interneurons and their synaptic populations have been explicitly compared with those in the retina of vertebrates [2], [3]. Like the latter, photoreceptors of two functional classes innervate the fly's optic lobe. These arise from an array of ommatidia in the overlying compound eye, each with a small, fixed complement of cells identified definitively in the fruit fly *Drosophila melanogaster* [4] and containing eight photoreceptor neurons. The six outer of these (R1–R6) terminate in the lamina [2], [5], while two central cells, R7 and R8, penetrate the lamina and innervate the second neuropil, the medulla [6]. In the lamina the axon terminals of R1–R6 provide synaptic input upon first-order interneurons grouped in cylindrical modules called cartridges [7], [8]. Like the ommatidia that innervate them, these too are of determinate composition; each cartridge comprise the six R1–R6 terminals and a fixed set of lamina neurons, one of each type, with the axons of R7 and R8 occupying a position satellite to these, as reported from electron microscopy for *Drosophila* [9]. The neuron types and their synaptic connections in a cartridge have been described by various techniques in the house fly *Musca domestica* and other larger fly species [2], [7], [8], [10], [11] as well as in the fruit fly *Drosophila melanogaster* [9], [12]. For the lamina of *Drosophila*, the synaptic contacts [9] and their numbers [13], as well as the circuits these constitute, have all been reported for the R1–R6 photoreceptor terminals and 11 major types of interneuron. The neuronal organization of the lamina is characterized by a geometrical precision of the arrangement of its neuronal elements into cartridges. As a result the identification of specific neurons has been greatly facilitated, both at the light and electron microscopical levels. Thus, the *Drosophila* lamina has become an excellent system for the analysis of the genetic regulation of many aspects of synaptic function, plasticity and synaptogenesis (see [14], [15], [16], [17], [18], [19], [20], [21]).

In parallel with the structural analyses of the lamina's synaptic circuits, which are most complete for *Drosophila*, the electrophysiological properties of lamina neurons are reported but mostly from larger fly species (e.g. [22], [23] [24], [25], [26], [27], [28], [29], [30]). Together, these reveal visual phenomena such as spatial summation and amplification of visual signals, lateral inhibition, light adaptation, and even peripheral substrates for movement detection and colour coding (reviewed in [31]). By contrast, only limited electrophysiological data are available for lamina neurons in *Drosophila* ([32], [33]).

In contrast to the extensive anatomical and electrophysiological investigations, we have little information about the neurotransmitters in the lamina of flies (see [34], [35]). It is clear that fly photoreceptors use histamine as their neurotransmitter [34], [36], [37], [38], [39], [40]. When released from photoreceptor synapses histamine acts as a fast neurotransmitter at ligand-gated chloride channels on postsynapic lamina interneurons [36], which include L1–L3 [41]. There is also immunocytochemical evidence for GABA in two types of small field centrifugal interneurons, C2 and C3 [42], [43], [44], [45]. This evidence is based on several antisera to GABA and antisera to the biosynthetic enzyme glutamic acid decarboxylase (GAD). Some reports indicate the immunolabeling of lamina monopolar cells (first-order interneurons) with antisera to glutamate, in flies [46], [47] and honeybees [48]. In *Drosophila*, these cells also label with an antibody against choline acetyltransferase (ChAT), the biosynthetic enzyme of acetylcholine [49], which is encoded by the gene *Cha* [50]. *Cha* transcript has also been found by *in situ* hybridization in cell bodies of lamina monopolar neurons [51]. Finally, fly amacrine cells are reported to express glutamate immunoreactivity [47]. Clearly there is some uncertainty in these reports. Some describe tentative identifications of lamina neurons, while in others the antisera used may identify a substance (e.g. glutamate) that is present only as a metabolic intermediate; some studies also do not include *Drosophila*. Thus, for *Drosophila* there is a need to investigate the lamina further with respect to these classical neurotransmitters.

Here we applied immunocytochemistry to the lamina of *Drosophila* to identify neurotransmitters or associated molecules important for neurotransmitter function, including corresponding receptors proteins. Examination of these markers was combined with use of the Gal4-UAS system [52] to drive expression of green fluorescent protein (GFP) in specific neuron populations of the lamina. The focus of our investigation is on neurons expressing markers for acetylcholine, glutamate, GABA, and some of their receptors.

## Materials and Methods

### Fly strains

We used adult wild type *Drosophila melanogaster* (Oregon R or *w^1118^* strains) for basic immunocytochemistry. For correlation with various neuronal phenotypes we performed immunocytochemistry on a variety of Gal4 lines crossed with UAS-GFP, as specified below. L2 monopolar interneurons were visualized by the 21D-Gal4 driver [53] (from Tomas Raabe, University Würtzburg, Germany). C3 neurons were identified by 5-6-8/CyO;TM2/TM6B-Gal4 (abbreviated 5-6-8-Gal4; from Larry Zipursky, Howard Hughes Medical Institute at UCLA, Los Angeles, CA). Other Gal-4 lines used were: *rdl*-Gal4 (4.7 kb upstream *rdl*-gene; from Julie Simpson, Howard Hughes Medical Institute, Janelia Farm, VA), *Cha*-Gal4 [54] (from Bloomington Stock Center at Indiana University, Bloomington, IN), and OK371-Gal4 ([55], from Hermann Aberle, University of Münster, Germany). These were used to visualize expression of the GABA_A_ receptor subunit RDL, choline acetyltransferase (*Cha*), and vesicular glutamate transporter (vGluT) gene products, respectively. To visualize Gal4-expression with GFP, we crossed these lines with flies expressing UAS-mCD8-GFP (from Bloomington Stock Center). Presynaptic sites were visualized by driving a neuronal synaptobrevin-GFP fusion line (w[*];P{w[!mC] # UAS-*nsyb.egfp*}2; Bloomington stock center) with either the OK371- or 21D-Gal4 lines (see [56], [57]).

### Antisera

Several antisera were used to detect neurotransmitters and other signaling components in the lamina. The antisera and their corresponding antigens are listed separately (Table 1). Antisrum specificities have been carried out for all antisera in earlier publications (listed in Table 1). A comprehensive description of antiserum production and specificity tests is given below.

**Table 1:** Antisera used for immunocytochemistry.

Antiserum	antigen	fixation	dilution	source (references)
**Glutamate** rabbit polyclonal	L-glutamate conjugated to KLH with glutaraldehyde (GA)	Zamboni's fixative	1∶10,000	Arnel. Products, New York, NY Cat. no. 1766 [62]
**Glutamate** mouse monoclonal	L-glutamate conjugated to KLH with GA	Zamboni	1∶5000	Sigma, Cat. no. G9282 [63]
**DmGluRA** #7G11 mouse monoclonal	*Drosophila* metabotropic glutamate receptor A (recombinant protein)	Zamboni	1∶10	European Molecular Biology Laboratory, Heidelberg, Germany [58]
**DvGluT** C-term rabbit polyclonal	*Drosophila* vesicular glutamate transporter (peptide sequence)	Zamboni, Bouin 4% PFA	1∶10,000	from Dr. A. DiAntonio, University of California, LA [61]
**DvGluT** C-term rabbit polyclonal	*Drosophila* vesicular glutamate transporter (amino acids 561–632)	Zamboni, 4% PFA	1∶1000	from Dr. H. Aberle, University of Münster, Germany [55]
**DvGluT** N-term rabbit polyclonal	*Drosophila* vesicular glutamate transporter (amino acids 21–87)	Zamboni, 4% PFA	1∶1000	from Dr. H. Aberle, Münster University, Germany [55]
**DvGluT** C-term affinity purified rabbit polyclonal	*Drosophila* vesicular glutamate transporter (amino acids 561–632)	Zamboni, 4% PFA	1∶500	from Dr. H. Aberle, Münster University, Germany [55]
**DLG** mouse monoclonal	Discs large protein (recombinant protein, PDZ2 domain )	4% PFA	1∶2000	Developmental Study Hybridoma Bank, NICHD, Iowa [104]
**GABA** #A2052 rabbit polyclonal	γ-aminobutyric acid (GABA-BSA)	4% PFA	1∶2000	Sigma-Aldrich [65]
**GAD-1** rabbit polyclonal	Glutamic acid decarboxylase-1 (purified protein)	Zamboni, Boiun	1∶1000	from Dr. F. R. Jackson [66], [67]
**GABA_B_R2** rabbit polyclonal	GABA_B_ receptor 2 (peptide sequence)	4% PFA	1∶16,000	[65]
**GFP** mAb 3E6 mouse monoclonal	Green fluorescent protein from *Aequorea victoria* (purified protein)	4% PFA, Zamboni, Bouin	1∶1000	Molecular Probes, Leiden, Netherlands
**ChAT** 4B1 mouse monoclonal	Choline acetyltransferase (recombinant protein)	4% PFA	1∶1000	Developmental Study Hybridoma Bank [69]
**NMDA1** subunit mab363 mouse monoclonal	Ionotropic glutamate receptor (mammalian) (recombinant protein)	Bouin	1∶500	Chemicon, Temecula, CA [47]
**RDL** subunit N-term rabbit polyclonal	*Drosophila* ionotropic GABA receptor (peptide sequence)	Zamboni, Bouin	1∶40.000	[68]
**vAChT** C-term Rabbit polyclonal	*Drosophila* vesicular acetylcholine transporter (amino acids 441–546)	4% PFA	1∶1000	from T. Kitamoto [73]

#### DmGluRA

The mouse monoclonal antibody to DmGluRA, 7G11 ([58]; purchased from European Molecular Biology Laboratory, Heidelberg, Germany) was raised against recombinant receptor protein that was purified to homogeneity [58], [59]. Specificity of 7G11 was tested by expressing DmGluRA in a baculovirus-insect cell system and testing cell extract by western blotting [59]. The 7G11 antibody was also tested on Western blots of head extracts of *Drosophila* controls (2b) and DmGluRA mutants (112b) showing loss of staining in mutants [60].

#### DvGluT

The Rabbit anti-DvGluT (*Drosophila* vesicular glutamate transporter; kind gift from Dr. A. DiAntonio, Washington University School of Medicine, St. Louis, MO; [61]) was raised against a C-terminal peptide (CQMPSYDPQGYQQQ) of the *Drosophila* vGluT, affinity purified and characterized by western blotting and by its detection of transgenically expressed vGluT [61].Two other polyclonal rabbit antisera to the *Drosophila* vGluT were raised against the C-terminus (amino acids 561–632) and N-terminus (amino acids 21–87) of the transporter protein, respectively. The C-terminus antiserum was affinity purified. Both antisera were kindly provided by Dr. H. Aberle (University of Münster, Germany; [55]). In *Drosophila* embryos homozygous for a small deficiency that removes the vGluT gene Mahr and Aberle [55] did not observe immunolabeling. They also found a good match between the immunolabeling obtained with the two vGluT antisera (indistinguishable from each other) to in situ hybridization and the OK371 (vGluT-Gal4) expression pattern.

#### Glutamate

We used two antibodies both raised against L-glutamate conjugated to keyhole limpet hemocyanin (KLH) with glutaraldehyde: a rabbit polyclonal (Cat. no. 1766; Arnel Products, New York, NY) raised by Hepler et al. [62]; and a mouse monoclonal (Cat. no. G9282; Sigma, St. Louis, MO) raised by Madl et al. [63]. The specificity of both antibodies in fly tissues is revealed because both gave similar labeling patterns in *Drosophila* to an antibody against vGluT (above), and both immunolabeled the same cells in two other species of fly (*Musca*, *Calliphora*) that they labeled in *Drosophila*.

#### GABA

We used a commercial antiserum to GABA (Sigma; Cat. No. A2052) that was raised to GABA-bovine serum albumin (BSA) conjugate and then affinity immunopurified by the manufacturers. The GABA antiserum was characterized by dot-blot immunoassay by the manufacturers and was previously applied to *Drosophila* brain [64], [65].

#### GAD-1

Antiserum to full-length gel-purified *Drosophila* GAD1 protein was raised in rabbit (kind gift from Dr. F.R. Jackson; [66], [67]. This antiserum has been previously characterized by Featherstone et al. [67] by Western blotting (recognizes a 57-kDa band, as expected) and by demonstrating the absence of labeling of tissue in a homozygous mutant lacking the *gad1* gene.

#### GABA_B_R2

Production of antisera to GABABR2 was described previously [65]. In brief, three antisera were raised in rabbits against a sequence (CLNDDIVRLSAPPVRREMPS) of the C-terminus of the receptor protein conjugated to KLH. These antisera were characterized by ELISA, Western blotting and with standard pre-adsorption tests [65]. In addition, preimmune sera from the rabbits were collected prior to immunization and used for immunocytochemistry and Western blotting as controls. The best antiserum (code B7873/3) was used here.

#### RDL

For The GABA_A_ receptor subunit RDL we synthesized a C-terminal peptide sequence: CLHVSDVVADDLVLLGEE, which was coupled to *Limulus* hemocyanine (LPH) via the N-terminal cysteine. The best RDL antiserum (code 7385) producedin rabbit was characterized by Western blotting and immunocytochemistry, by pre-adsorption with peptide used for immunization, and by tests of preimmune serum [68].

#### ChAT

A mouse monoclonal antibody to recombinant ChAT protein (Code 4B1; [69]) was purchased from the Developmental Study Hybridoma Bank. This antibody was characterized by pre-adsortption with crude recombinant ChAT [69], [70] and the labeling pattern in *Drosophila* confirmed by in situ hydridization and LacZ expression (see [49]).

#### NMDAR1

Mouse monoclonal antibodies raised against the rat NMDAR1 (mab363) were purchased from Chemicon (Temecula, CA) who performed specificity test of the antibodies (no cross reactivity with other NMDA receptors). The antiserum was raised against a recombinant fusion protein containing the amino acids 660–811 of rat NMDAR1 [71]. The NMDAR1 protein displays 46% amino acid identity to a *D. melanogaster* NMDA-like protein [72]. This antibody was previously utilized on the lamina of flies and honeybees by Sinakevitch and Strausfeld [47].

#### vAChT

A rabbit polyclonal antiserum to vAChT was raised against amino acids 441–546 of the protein [73]. The antiserum was characterized in western blots of extract from wild type (band with *M*r of 65 kD) as well as *vacht*- (vesicular acetylcholine transporter) and *Cha*-mutant flies [73], [74].

#### vGAT

Antiserum was raised in rabbits to a peptide sequence (N-terminal amino acids 24–38) of the putative *Drosophila* vesicular GABA transporter (vGAT; CG8394). The peptide was synthesized with a cysteine *C*QTARQQIPERKDYEQamide for directed conjugation to maleimid-coupled KLH at the N-terminal. The best vGAT antiserum (code 1061) was affinity immunopurified. This antiserum was characterized by Western blotting, pre-adsorption with the peptide used for immunization, and tests of preimmune serum [68].

#### GFP

A mouse monoclonal antibody to GFP (mAb 3E6; code #A-11120; Molecular Probes, Leiden, Netherlands) was used at 1∶1000 for amplifying the GFP signal in some specimens. This antibody was raised against GFP purified from the jellyfish *Aequorea victoria* and characterized by the manufacturer; it produces no immunolabeling in wild type *Drosophila* CNS and thus only amplifies the GFP fluorescence.

### Immunocytochemistry

For glutamate immunolabeling, brains were dissected out of the head capsule in modified Zamboni's fixative (4% paraformaldehyde, 1.6% glutaraldehyde, 0.2% saturated picric acid, in 0.1M sodium phosphate buffer, pH 7.4) and left for between 1 h and overnight at 4°C. They were washed in sodium phosphate buffered saline (PBS), and then sectioned at 50–80 µm slices on a Vibratome. The sections were washed in PBS, blocked with normal goat serum (NGS), transferred to 0.5% Triton X-100 in PBS for 30 min prior to primary antibody incubation, and then and incubated overnight at 8°C in one of two glutamate antisera (Table 1), a rabbit polyclonal antibody at a dilution of 1∶10,000, or a monoclonal antibody at a dilution of 1∶5000. After the primary antibody, the tissue was washed several times in PBS, and then incubated with the corresponding secondary antibody (goat anti-rabbit, goat anti-mouse: Jackson ImmunoResearch Labs, West Grove, PA) conjugated to a fluorochrome (Cy3 or FITC).

For all other immunocytochemistry the adult fly heads were dissected in PBS-TX (0.01M phosphate-buffered saline with 0.5% Triton X-100, pH 7.2) before fixation. For DmGluRA, DvGluT and DLG immunolabeling, opened heads were fixed 2 h in freshly prepared ice-cold Zamboni's fixative (4% paraformaldehyde and 0.5% picric acid in 0.1 M phosphate buffer, pH 7.2) or 4% paraformaldehyde in 0.1 M sodium phosphate buffer (PB) at pH 7.4. For GABA_B_R2 immunolabeling, fly heads were fixed for 2 h in ice-cold 4% paraformaldehyde in 0.1 M PB (pH 7.2). For GABA and GAD immunolabeling, the heads were fixed in freshly prepared ice-cold Bouin's fixative for 30 min (tissues were also fixed in Zamboni's fixative to obtain better GFP preservation). For ChAT and vAChT immunolabeling, adult fly heads were fixed for 2 h in ice-cold 4% paraformaldehyde (pH 7.4) or in Zamboni's fixative. Additional labeling with anti-GFP was necessary to amplify GFP fluorescence partly quenched after Bouin fixation.

Tissues were thoroughly washed with 0.1 M PB and incubated overnight at 4°C in 20% sucrose in 0.1 M PB. 20 µm thick sections of the head were cut on a Leitz 1720 Cryostat at −23°C and collected on chromalum-gelatin–covered microscope slides. After washing in PBS-TX, tissues were incubated with primary antibodies in 4°C overnight or for 48 h. Brains were washed in PBS-TX at room temperature (about 22°C) and incubated with fluorophore-tagged secondary antibodies (Cy2- or Cy3-tagged IgGs, raised in goat; Jackson ImmunoResearch) diluted 1∶1000, either overnight at 4°C or 2 h at room temperature (around 22°C). After washing in PBS-TX and rinsing in 0.01 M PBS, tissue was mounted under a coverslip in 20% glycerol in 0.01 M PBS.

### Pre-embedding immuno-electron microscopy

We also used both wild-type and white eye mutants for electron microscopy of preparations immunolabeled for glutamate by the pre-embedding method using the polyclonal rabbit anti-glutamate [62]. Tissue incubated as above in this primary antibody was next incubated in a biotinylated goat anti-rabbit antibody (Vector Labs) and then in a solution of peroxidase conjugated Avidin Biotin Complex (ABC complex, Vector Labs). Labeling was detected with 3,3′-diaminobenzidine (DAB) as the substrate. The sections were then osmicated, dehydrated in graded ethanol series, changed into propylene oxide and then flat-embedded between Aclar sheets (Ted Pella Inc, Redding, CA) in Poly/Bed 812 resin. The tissue was sliced at 80 µm using a Vibratome and selected slices subsequently resectioned at 60 nm for electron microscopy. Ultrathin sections were then viewed at 60 kV with a Philips 201 C electron microscope, photographed on 35 mm film at primary magnifications of between 5,000 and 20,000, and the prints then labeled and scanned.

### Imaging

For glutamate-immunolabeled specimens, Vibratome slices were mounted in Vectashield (Vector Labs, Burlingame, CA) and viewed with a Zeiss LSM 410 confocal microscope (Zeiss, Jena, Germany). All other specimens were imaged with a Zeiss LSM 510 confocal microscope. Confocal images were obtained at an optical section thickness from 0.1–0.35 µm and were processed with Zeiss LSM software and edited for contrast and brightness in Adobe Photoshop CS3 Extended version 10.0.

## Results

**Figure 1:**
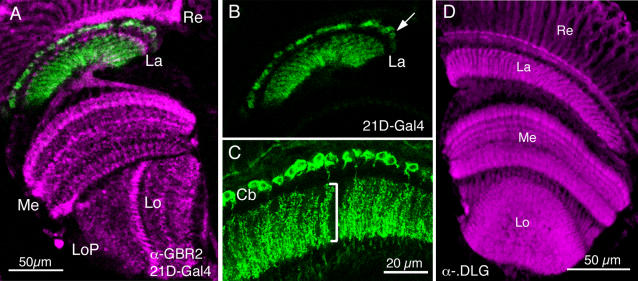
The optic lobe of *Drosophila melanogaster.* A Horizontal section showing part of the retina and neuropil layers of the visual system (labeling with antiserum to GABA_B_R2): retina (Re) with photoreceptors, lamina neuropil (La) connected with the medulla neuropil (Me) via the first optic chiasma. Central to these are two neuropil layers: the lobula (Lo) and lobula plate (LoP). Scale bar = 20 µm. B The same section revealing lamina (La) with GFP-labeled L2 monopolar interneurons (21D-Gal4) with distal cell bodies (arrow). C Enlargement of L2 monopolar cells, with a single row of cell bodies (Cb) located in the overlying lamina cortex between retina and lamina neuropil. Bracket indicates depth of extensive L2 spines in synaptic neuropil. Scale bar = 10 µm. D Frontal section of the optic lobe immunolabeled with DLG antiserum. This antiserum visualizes structures within photoreceptors terminating in the lamina and also neuronal structures in the stratified neuropil of the medulla. Magnification same as in A.

**Figure 2:**
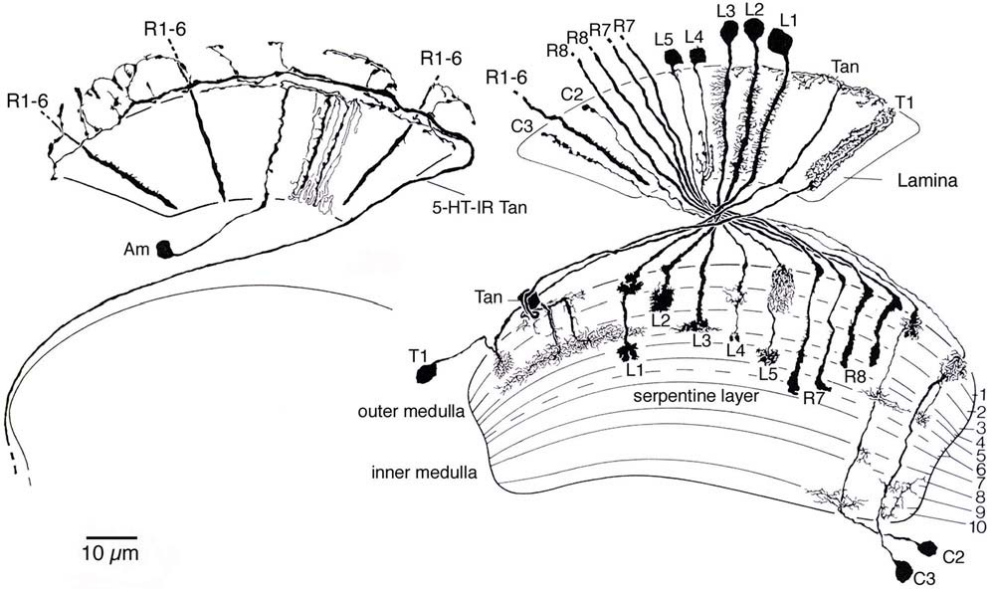
Neuron types in the lamina of *Drosophila.* The neurons were revealed by Golgi silver impregnation in *Drosophila melanogaster* (The figure was modified from Meinertzhagen and O'Neil [9], after Fischbach and Dittrich [12]). A wide-field amacrine neuron (Am, designated Lai by Fischbach and Dittrich [12]) and wide-field serotonin-immunoreactive tangential neuron (5-HT-IR Tan). B The different types of narrow-field neurons of the lamina (and one wide-field neuron: Tan, designated Lat by Fischbach and Dittrich [12]) and their relationships in the 10 medulla strata comprise: R1–R6, terminate in the lamina; R7 and R8, in the medulla; L1–L5 lamina monopolar neurons; C2 and C3 narrow-field centrifugal neurons; T1, a narrow-field centripetal neuron with input in the lamina; and Tan (originally called La wf1), a wide-field tangential neuron. A second type of tangential neuron, La wf 2, illustrated by Fischbach and Dittrich [12], is not incorporated in this figure.

The optic lobe of *Drosophila* consists of four neuropil regions located beneath the retina: the lamina, medulla, lobula and lobula plate (Fig. 1A). Each of these neuropils exhibits a columnar organization that derives from the pattern of photoreceptor innervation from the ommatidia of the overlying retina. R1–R6, the six outer of eight photoreceptor neurons in each ommatidium, terminate in the lamina in columnar modules termed cartridges [5], [7], while the two inner neurons, R7 and R8, penetrate the lamina and innervate the distal strata of columns in the medulla (see Fig. 2B). The optic lobe neuropils are also stratified (Figs. 1A,D, and 2B), the result of overlap between stratum-specific terminals of (1) columnar centripetal neurons (those running from periphery to center), (2) columnar centrifugal neurons (those running in the opposite direction); and lateral arborizations (dendrites or collaterals) of (3) various columnar neuronal elements and (4) tangentially oriented wide-field branches of non-columnar neurons (see [12], [75]. Some neurons do not display a pronounced stratified organization within the lamina. For example the L2 monopolar neurons form uniform arrangements of short, radially-directed dendritic spines throughout the depth of the lamina neuropil (Figs. 1B,C, and 2B). In contrast to their processing counterparts in the vertebrate retina, a distinctive feature of insect neurons is that they have their cell bodies located in a cortex surrounding the synaptic neuropil (Fig. 1C). Thus all interneurons referred to in this report have cell bodies in the lamina cortex, or in a cortex of the deeper optic lobe.

**Figure 3:**
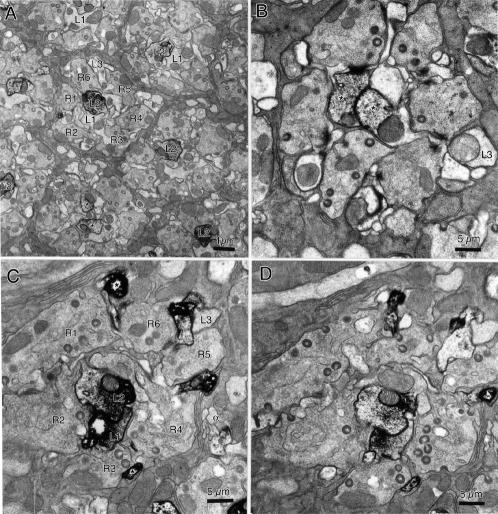
Glutamate immuno-EM of lamina cartridges in *Drosophila* exhibits a range of labeling patterns. A Preparation in which only profiles of L2 exhibit immunoreactivity. One cartridge has two profiles (asterisks), thought to derive from the single L2 axon and one of its basal dendrites. Confirmation of their common origin would require serial sections. Scale bar: 1.0 µm. B Two profiles (asterisks) of L1 and L2 axons, identifiable as a pair but not individually, show clear immunolabeling; their dendritic spines in this preparation do not, the label stopping at the base of a dendrite (arrowhead), nor does the profile of L3. C Heavily labeled profiles (asterisks), insinuated between R1–R6, lack connection to the two immunolabeled axon profiles of L1 and L2, and are therefore identified as α-process of amacrine cells. Unlike L1 and L2, which show clear immunolabeling, the axon profile of L3 lacks label. R1–R6 identified with respect to profiles of L3 and amacrine cell axons (α). D Similar labeling pattern as in C. Scale bar: 0.5 µm.

**Figure 4:**
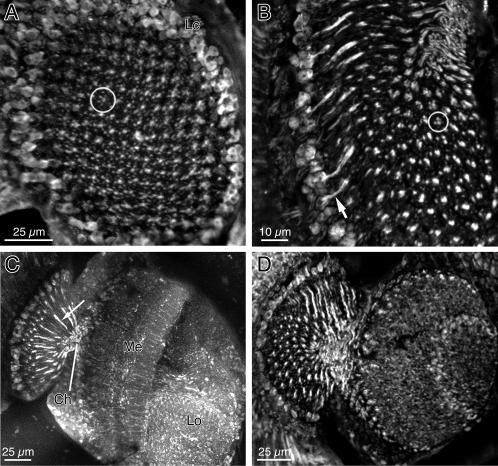
Confocal examination of glutamate-like immunoreactivity in the optic lobes of *Drosophila.* A Tangential view of the distal lamina cutting the array of cartridges to reveal their regular labeling pattern in the neuropile, surrounded by at least two rows of immunoreactive somata in the lamina cortex (Lc). Each cartridge cross-section (circle) comprises two axial L-cell profiles surrounded by smaller α-profiles of amacrine cells. B At a deeper level to that in A, each cartridge profile contains two or three glutamate-like immunoreactive axial profiles (circle) with immunoreactive fibers extending from monopolar somata (arrow). C Frontal section, showing coarse, longitudinally sectioned immunoreactive axon profiles (arrow) in the lamina, extending into the chiasma (Ch), and columnar and tangential immunoreactive elements in the medulla (Me) and lobula neuropils. D Frontal section at one edge of the lamina and medulla cuts these neuropiles obliquely.

To facilitate interpretation of the immunolabeling and GFP expression patterns in the lamina and distal medulla we first briefly present the neuron types of the *Drosophila* lamina. The neuronal morphologies depicted in Fig. 2 are based on analyses of a large number of Golgi impregnations of *Drosophila* [12]. There are 3 types of photoreceptor axon and 11 types of interneuron associated with the lamina. Most of these are columnar, with an axon oriented parallel to the main axis of the visual columns, thus establishing the retinotopic organization of the optic lobe. All the interneurons, except the wide-field elements (amacrines and tangential neurons), are readily distinguished and morphological counterparts have been identified in other fly species [2], [12], [76] that have been reasoned to be evolutionary homologues [77]. Together, the columnar elements form a bundle of invariant pattern and composition, the axon of each contributing a distinct profile to the cartridge cross section (Fig. 3A, 4A,B).

There is, however, some ambiguity with respect to amacrine and tangential neurons. This is important to point out in order to accurately interpret our immunolabeling and Gal4-GFP expression patterns (see later sections). Fig. 2A depicts one type of amacrine (Am) and one of two types of tangential neurons (5-HT-IR Tan). The other (Tan; designated La wf1 by Fischbach and Dittrich [12]) has arborizations in the distal synaptic layer of the lamina (Fig. 2B), while 5-HT-IR Tan (designated Lat by [12]), has all its varicose processes in a layer distal to the lamina neuropil, and is known in *Drosophila* and larger flies to react with antisera to serotonin (see [35]). In the paper by Fischbach and Dittrich [12]a possible third type of tangential neuron (La wf2) is depicted (in their Fig. 24F). This also has processes reaching into the distal lamina, but its morphology differs from that of Tan (their La wf1). La wf2 has tangential branches with large boutons hanging down into the lamina neuropil. Only one type of amacrine (Am; designated Lai by Fischbach and Dittrich [12]) was described in *Drosophila*, with tangential processes sprouting characteristic α-processes running between the R1-R6 terminals in the cartridges. These make many synapses [13]. However, in other flies a second type (Am2) has been noted [30], [76], which seems to lack the α -processes and have all its processes distal to the lamina neuropil.

It is noteworthy that the distinguishing morphology of many types of lamina neuron can best be detected in the medulla (Fig. 2B), which is also modular in organization (Fig. 4A,B). Thus, for example, L1–L5 and C2 and C3, each have a characteristic terminal or arborization in a distinct set of medulla strata.

In the following sections we describe the different neurotransmitter systems as revealed by different markers for acetylcholine-, glutamate- and GABA-associated molecules. For coherence we have organized the figures according to the major neuron types in the lamina, with the result that a few figures fall out of numerical order in the text.

### Acetylcholine signaling components in the lamina

**Figure 5:**
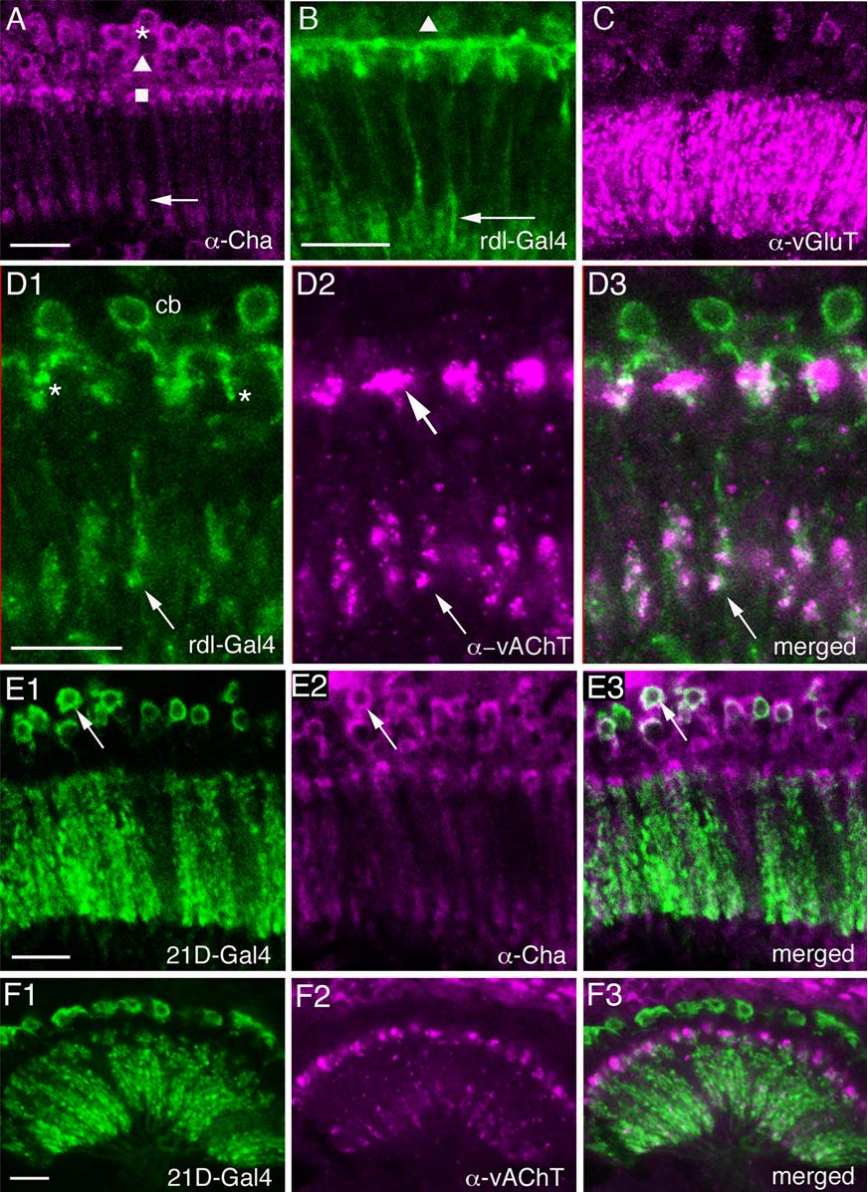
Monopolar neurons in the lamina labeled with different antisera or Gal4 lines. A ChAT immunoreactive lamina neurons. Asterisk: layer with large monopolar cells (L1–L3); triangle: layer with small monopolar cells (L4 and L5); square: layer with processes of *Cha*-Tan neurons (see Fig. 8 and 9). Arrow labels level of branching of L4 neurons in the proximal lamina. Scale bar = 10 µm (for all images, except panel B). B GFP expression in lamina driven by the *rdl*-Gal4 reveals L4 neurons. Triangle: L4 cell body; arrow: characteristic branching of L4 collaterals in the proximal lamina. GFP is also seen in branches of a wide-field tangential neuron, in the distal lamina. Scale bar = 10 µm. C Weak immunolabeling in cell bodies of large monopolar neurons with antiserum to vGluT. Strong immunolabeling in the lamina neuropil is seen in processes of amacrine neurons. D1–3 Distributions of vesicular acetylcholine transporter (vAChT) immunoreactivity and *rdl*-Gal4 driven GFP expression co-localize to arborizations of the L4 neurons (arrow) in the proximal lamina, but not in their cell bodies (cb) and not in processes of *rdl*-Tan neurons (asterisk in D1) in the distal lamina. However, vAChT immunoreactivity is seen in enlarged boutons of another tangential neuron in this dorsal layer (large arrow). Scale bar = 5 µm. E1–3 Cell bodies of L2 monopolar neurons are ChAT immunoreactive, revealed by 21D-Gal4 driven GFP (green) in L2 neurons labeled with anti-ChAT (α-Cha; magenta). Co-localization of label is seen in cell bodies, but not clearly in their neurites. Scale bar = 10 µm. F1–3 Anti-vAChT labeling (α-vAChT; magenta) is not co-localized in L2 monopolar cells displayed by GFP driven by 21D-Gal4. Scale bar = 10 µm.

Acetylcholine is a major excitatory neurotransmitter in *Drosophila* and other insects [49], [78], [79]. Choline acetyltransferase (ChAT) and the vesicular acetylcholine transporter (vAChT) are essential for cholinergic neurotransmission and antisera to these proteins are phenotypic markers for cholinergic neurons [54], [73]. Several papers have used ChAT antisera or *in situ* hybridization for *Cha* transcript to localize presumed cholinergic neurons to the *Drosophila* visual system [49], [51], [70], [80], but to our knowledge none has yet employed vAChT antiserum to this part of the brain. We examined the *Drosophila* lamina with antisera to both proteins. ChAT-immunolabeling reveals several types of lamina neuron (Fig. 5A). The cell bodies of large and small monopolar neurons are ChAT-immunolabeled (Fig. 5A,E), and what appear to be the axons and tripartite lamina collaterals of L4 monopolar neurons also react with ChAT antiserum (Fig 5A, see also 5B). The axons of other monopolar neurons were not seen. Using 21D-Gal4 to drive GFP in L2 cells we could also show that anti-ChAT labeled L2 cell bodies, but no immunolabeling was visible in their dendritic processes in the lamina (Fig. 5E).

**Figure 6:**
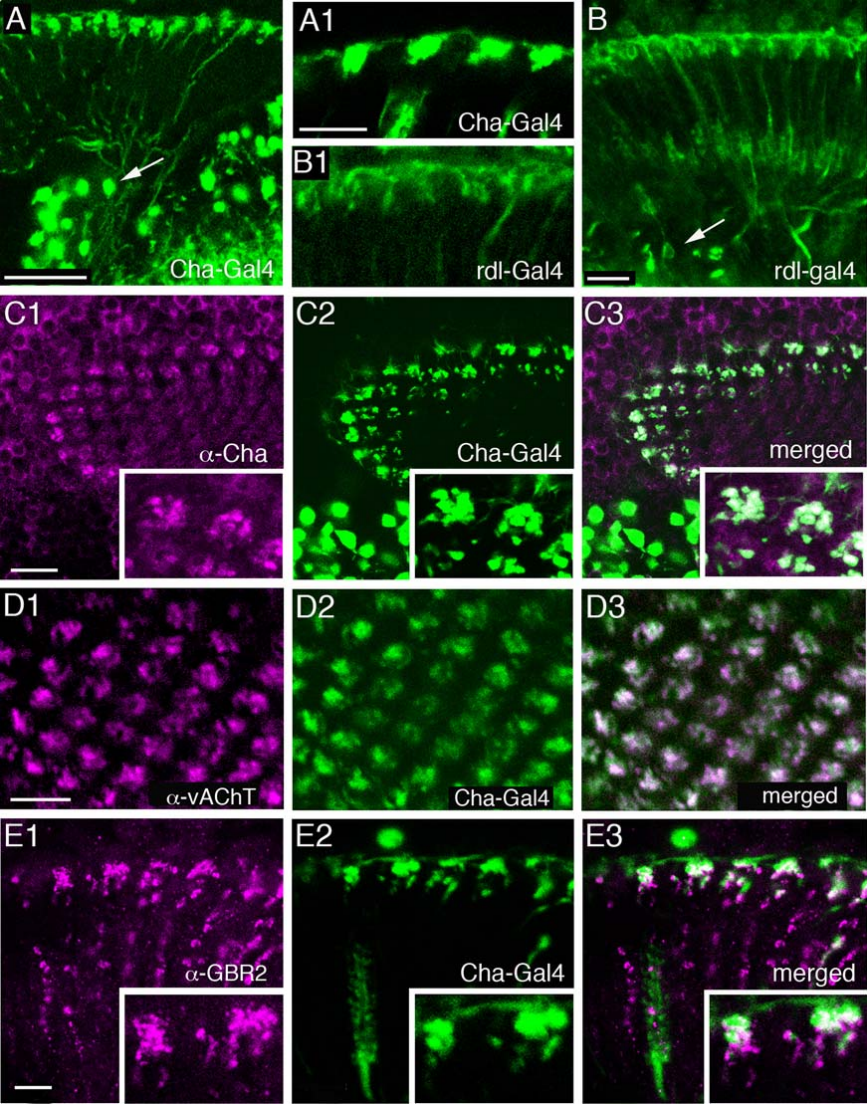
Tangential and amacrine neurons in lamina, and markers for GABA, glutamate and acetylcholine signaling. A GFP expression driven by *Cha*-Gal4 reveals putative choline acetyltransferase containing lamina neurons. GFP expression in cell bodies near the medulla (arrow), and lamina morphology together suggests that *Cha*-Gal4 reports Tan neurons (La wf1) in the lamina. Scale bar = 20 µm. A1 Magnification of the putative Tan varicosities in the distal lamina. Scale bar = 10 µm. B GFP expression in Rdl-Gal4 reveals tangential neurons in lamina with cell bodies localized above medulla (arrow). Scale bar 10 µm. B1 Enlarged view of the Rdl-Gal4-expressing lamina neurons. Scale as in A1. C(1–3) Distribution of ChAT immunoreactivity (C1, α-Cha; magenta), in relation to *Cha*-Gal4 driven GFP (C2, green) in lamina cross section. Co-localization (C3) is seen in the distal rosette-like structures (see magnifications in insets). Scale bar = 10 µm. D (1–3) Cross section of lamina showing co-localization of GFP in *Cha*-Gal4 and anti-vAChT (D1, α-vAChT: magenta) in distal boutons of *Cha*-Tan neurons. Scale bar = 10 µm. E (1–3) GABA_B_R2 immunoreactive neurons (E1, α-GBR2; magenta) in relation to *Cha*-Gal4 driven GFP in tangential neurons (E2, green). Close contacts and some co-localization (E3) between labels are seen (insets show magnified views), suggesting localization of GABA_B_R2 on these tangential neurons. Scale bar = 5 µm.

Additional to the cell bodies and presumed L4 processes, the ChAT antiserum also labeled enlarged boutons at the level of the C2 terminals (Fig. 5A, 6A,C). These structures seem to be associated with tangential neuronal elements having boutons in the distal lamina neuropil. Using *Cha*-Gal4 to drive GFP we obtained strong fluorescence in tangential neurons with similar boutons (Fig. 6A), but no labeling of any monopolar neurons. It is not clear whether these tangential processes seen with *Cha*-Gal4 are derived from the Tan tangential neurons (see Fig. 2B) or a novel type of tangential neurons (or even new amacrine neurons, like Am2 of other flies), both with more pronounced varicosities distal to the lamina neuropil than Tan. Arguing against the amacrine neuron possibility, the *Cha*-Gal4 expressing neurons appear to derive from neurons with axons projecting towards or even connecting to the medulla (Fig. 6A). Thus they are most likely to be a form of wide-field tangential neuron. For simplicity we will therefore refer to these neurons henceforth as *Cha*-Tan neurons. We found co-localization of *Cha*-Gal4 driven GFP and anti-ChAT immunolabeling in the tangential processes and enlarged boutons of these cells (Fig. 6C), but ChAT-immunolabeling was detected mainly in the GFP-labeled processes in the distal lamina, not in their cell bodies or axons (data not shown). Our immunocytochemistry thus confirms that the lamina neurons seen in the *Cha*-Gal4 reporter line actually express ChAT-immunoreactivity. We could exclude that the ChAT-immunolabeling in this distal layer is derived from C2 neurons, although, as we show later, C2 neurons may contact the *Cha*-expressing tangential neurons on these enlarged boutons. Seen in cross section, it appears that the overlapping *Cha*-Tan neurons form aggregates of boutons, each aggregate associated with an underlying cartridge (see section on GABA receptors).

The antiserum to the vAChT confirmed most of the ChAT-immunolabeling in the lamina. We could detect vAChT-immunolabeling in basal processes likely to represent collaterals of L4 neurons (Fig. 5D) and in the dilated boutons of the *Cha*-Tan neurons (Fig. 6D). Double-labeling with *rdl*-Gal4 and vAChT antiserum showed the close match between the two in the morphology of the L4-like profiles (Fig. 5D). This double-labeling also clearly showed the distinction between the *rdl*-Gal4 (described below) and *Cha*-Gal4 expressing tangential processes in the distal lamina. Whereas the *Cha*-Gal4 tangential profiles co-localize another acetylcholine marker, vAChT (Fig. 6D), the *rdl*-Gal4 processes did not (see Fig. 5D). Furthermore, the vAChT antiserum did not label neuronal cell bodies in the lamina cortex (Fig. 5D), and as a result we could not match the *rdl*-Gal4 signal with ChAT-immunolabeling in monopolar cell bodies, even those of L4 cells.

### Glutamate signaling components in the lamina

Immunocytochemistry has previously suggested the presence of glutamate in the large lamina monopolar neurons, L1 and L2, in the flies *Drosophila*, *Musca*, *Calliphora* and *Phaenicia sericata* [46], [47] as well as in type 1 amacrine neurons of the latter fly [47]. Here, we examined the *Drosophila* lamina for evidence of glutamate neurotransmission by applying antisera to two essential molecules, the neurotransmitter glutamate and the *Drosophila* vesicular glutamate transporter (vGluT). The presence of glutamate is a requirement for its candidacy as a neurotransmitter, but given the widespread availability of glutamate as an intermediary metabolite, this evidence alone is unacceptably weak. On the other hand, vGluT is required to load synaptic vesicles with glutamate and is a highly specific marker for sites of glutamate neurotransmission so that, for example, vGluT antisera label motoneuron varicosities [55], [61] that are known to utilize glutamate as a neurotransmitter [81].

#### Glutamate immunolabeling

To seek the presence of glutamate in the lamina, we examined the lamina from preparations sectioned in either a tangential (Fig. 4A,B) or frontal plane (Fig. 4C,D) or, in immuno-EM preparations, in a plane cut at a tangent to the lamina's surface (Fig. 3A), to reveal cross-sections of individual cartridges (Fig. 3B). Strong glutamate immunolabeling of monopolar cell profiles was apparent in all cartridges, and the corresponding terminals in the medulla.

The labeling pattern in *Drosophila* visible by confocal microscopy was substantially similar to, but varied in details from, that seen in two other fly species, the housefly *Musca domestica*, and the blowfly *Calliphora erythrocepha* (Figures S1, S2, and S3). A number of immunoreactive profiles were visible in single cross-sections of the cartridge, but the slender axon size *Drosophila* lamina cells gave some uncertainty in the exact determination of which profiles were axons and which dendrites. The small cartridge diameter relative to the somata of monopolar cells in the lamina cortex, and the short axon path between cortex and neuropil, made it particularly easy to identify the cell body fiber of immunoreactive monopolar cells (Fig. 4B). There were two rows of such somata above the cartridge (Fig. 4A). Similarly, it was easy to see the axons of monopolar cells extending into the chiasma (Fig. 4C). The deeper neuropiles showed qualitatively similar labeling patterns to those in the larger flies, but were not examined further.

For immuno-EM studies, we used a pre-embedding method with the polyclonal antiserum [62] This revealed a clear pattern of labeling that confirmed at higher resolution much of what was seen by confocal microscopy, and resolving the pattern of labeling of tiny profiles in *Drosophila*. From the enhanced resolution of the preparations we could also demonstrate that there was no difference in the labeling patterns in the lamina between preparations from wild-type flies, with red eyes, and mutant with white eyes. The consensus pattern was also highly consistent in all three fly species examined (Figures S1A–E).

The pattern of immuno-EM labeling in individual preparations varied somewhat. In some only a single monopolar cell axon profile, probably of L2 (Fig. 3A), was labeled. The basis for this identification was twofold. First, it was generally the larger of the axial monopolar cell profiles, as previously reported in a statistical sense [82], [83]. The same profile was labeled in surrounding cartridges, even if such a size difference was not seen in all. Second, we identified the profile by virtue of its position with respect to those of L3, between R5 and R6, and of a bundle of small amacrine cell fibers near R4 [9]. Such profiles did not accompany all cartridges however and were sometimes ambiguous, leaving some residual doubt about the identity of the labeled profile. Other preparations had the profiles of both L1 and L2 labeled (Fig. 3B), as was also seen in *Musca* (Figures S2, S3). Unlike the two other fly species, L3 was apparently not labeled in *Drosophila*. Cartridge profiles in the same preparation had the same immunolabeling patterns, so that variation was mostly between specimens.

In addition to axon profiles of L-cells, small immunoreactive profiles were visible between profiles of the R1–R6 terminals. These were especially clear in the cartridges of *Drosophila* (Fig. 3C,D) when labeled heavily with the pre-embedding method, compared with those of *Musca* (e.g. Figure S3B). Such locations are occupied by dendrites of both L1–L3 and amacrine cell alpha-processes that approach tetrad photoreceptor synapses [9]. In some preparations it was clear that immunolabel in the L-cell axon profiles disappeared at the base of the dendrites that arose from these (e.g. Fig. 3B: *Drosophila*; Figure S2A: *Musca*). The small labeled profiles between R1–R6 also never connected with the axon profiles of L1 and L2 (Fig. 3C,D). Both observations provide strong evidence that the immunolabeled profiles were instead those of the alpha-processes from amacrine cells. Corresponding somata of the amacrine cells were not examined.

#### *Drosophila* vGluT immunolabeling

**Figure 7:**
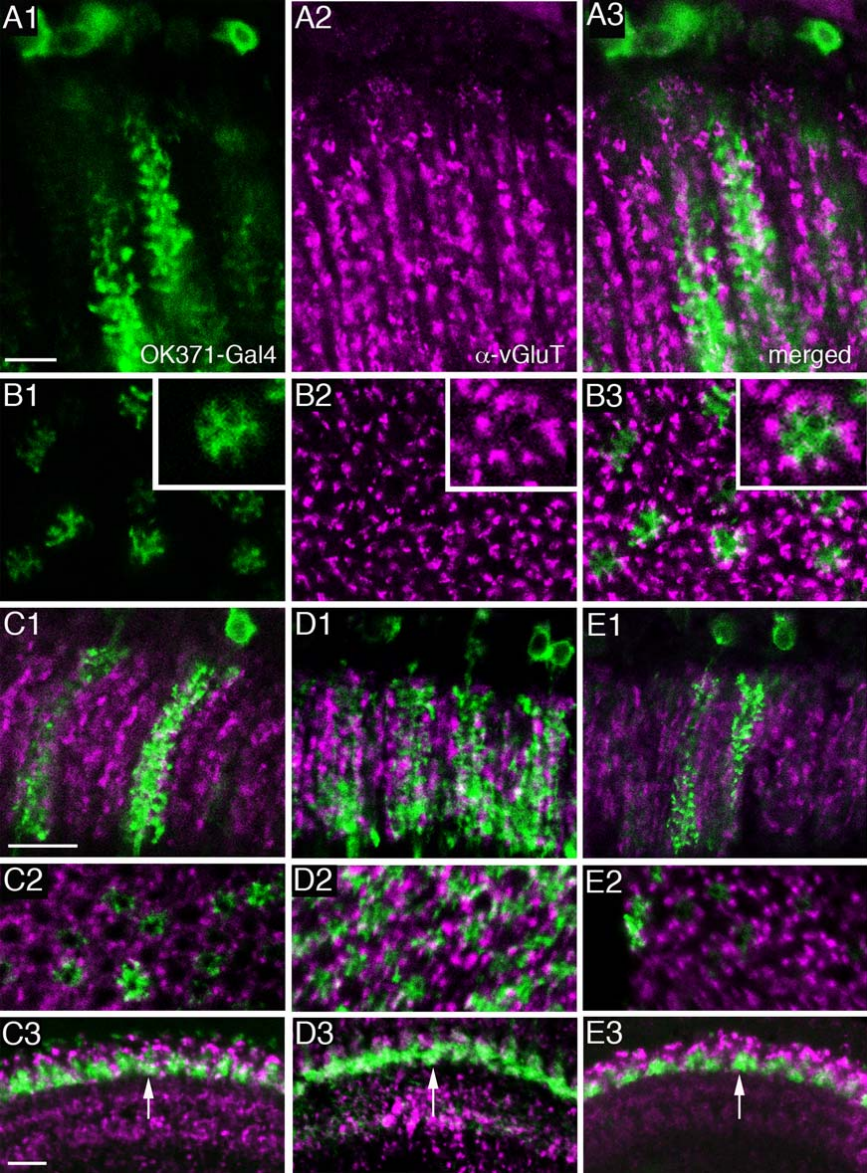
Distribution of vesicular glutamate transporter (vGluT) immunolabeling. Different antisera were combined with OK371-Gal4 driven GFP expression, which reports vGluT-expressing neurons. A1–3 Frontal sections of the lamina (vGluT antiserum) revealing lack of co-localization between vGluT immunolabeling (magenta) and GFP expression (A3 merged) in L2 monopolar cells (A1, green). Scale bar = 5 µm. B1–3 Cross-section of the same lamina region with enlargements in insets. B1 GFP in L2 neurons, B2 vGluT immunolabeling, B3 merged. Note that the vGluT immunolabels six structures surrounding the margin of the cartridge and at the extensions of L2 dendrites. C–E Similar structures labeled with three other antisera to vGluT. All vGluT antisera display the same immunolabeling in the lamina and medulla. C1–3 Affinity purified antiserum to vGluT (magenta) applied to OK371-Gal4 driven GFP (green). C1 Frontal section of lamina. Scale bar = 10 µm. C2 Cross-section of lamina (same magnification). C3 Frontal section of medulla showing that vGluT immunolabeling is not in GFP-labeled terminals of L2 cells in stratum M2 (arrow). Scale bar = 10 µm. D1–3 Similar structures labeled with antiserum to N-terminus of vGluT. Same scales as C. E1–3 Similar images using antiserum to the C-terminus of vGluT. Scales as in C.

To confirm that immunoreactivity to glutamate signified a capacity for glutamatergic transmission in the monopolar cells, we also applied four different antisera to the *Drosophila* vGluT and obtained identical labeling with each (Fig. 7). Strong vGluT immunolabeling was detected in profiles similar to α-processes of the amacrine neurons or possibly like β-processes of T1 neurons (Fig. 7; Figure S4). Weak vGluT immunolabeling of cell bodies was seen in the chiasma between the lamina and medulla, in a position corresponding to those of amacrine cells (Fig. 2A), but it was not possible to connect these to lamina processes (Figure S4A). The vGluT immunosignal in the lamina was mostly distinct from that seen with the OK371-Gal4 [55], representing vGluT promoter expression (Fig. 7A,B). The OK371-Gal4 drove GFP in smaller or larger populations of large monopolar neurons (Fig. 7A–E). Thus, instead of a complete co-localization of OK371 driven GFP and anti-vGluT expression, we saw neurons expressing vGluT lying adjacent to the GFP-labeled large monopolar neurons (Fig. 6A3,B3). In cross-section, six profiles in each cartridge expressed vGluT immunolabeling and surrounded the GFP labeled monopolar neurons (Fig. 7B,C2,D2,E2). To confirm the failure of vGluT immunolabeling to localize to processes of monopolar neurons, we investigated the relation between this label and OK371-driven GFP in terminals of monopolar neurons in the medulla (Fig. 7C3, D3 and E3). No clear co-localization was detectable. However, some vGluT-immunolabeling can be seen in cell bodies of large monopolar neurons (Fig. 5C) and we could not rule out low levels of vGluT immunolabeling in dendrites of monopolar cells that also express OK371-Gal4 (see Fig. 7A3,B3). Thus there is a lack of correspondence between data from the antisera and data from the Gal4 driver. OK371 expression indicates that at least the large monopolar cells express vGluT (*vglut*-promoter), just as they also contain glutamate, whereas at best the vGluT antisera only weakly label the corresponding cell bodies and tips of dendrites. On the other hand the vGluT immunolabeling seen probably in amacrine cell processes is not matched by a similar pattern of GFP-labeling for the vGluT promoter. Part of this discrepancy may reflect the different intraneuronal distribution of the markers: vGluT antibodies label predominantly presynaptic sites while GFP (cd8-GFP) expression is distributed in the plasma membrane throughout the neuron. There may also be very small amounts of highly localized vGluT protein in L1 and L2 compared with the surrounding amacrine cell processes. To investigate this possibility we used a neuronal synaptobrevin-GFP fusion (nsyb-gfp) to target GFP primarily to presynaptic sites (Figure S5). Using the 21D-Gal4 to drive nsyb-GFP resulted in fluorescence localized predominantly or exclusively to the medulla terminals of the L2 neurons (Figure S5A), but still no co-localization was seen with vGluT immunolabeling (Figure S5 B–D). Moreover with OK371-driven nsyb-GFP there was no co-localization to vGluT immunolabeling (Figure S5 E1–3). Finally, we cannot exclude that the OK371-Gal4 expression in neurons is incomplete because it lacks promoter/enhancer elements in the construct.

To reveal more clearly the relationship between vGluT-immunolabeled amacrine cell processes and the terminals of photoreceptors R1–R6, we used antibodies to Discs large (DLG) as a marker. The DLG protein is a membrane associated guanylate kinase (MAGUK) family protein located at the pre and postsynaptic area of functional glutamatergic synapses, at least in the *Drosophila* neuromuscular junction [84]. The vGluT immunolabeled structures are likely to be amacrine α-processes that seem to make contacts with DLG immunolabeled photoreceptors (Figure S4B1).

#### *Drosophila* GluR immunolabeling

As a further step, we also tried to localize glutamate receptors to lamina neurons using antisera to the *Drosophila* metabotropic glutamate receptor DmGluRA and one of the subunits of a mammalian ionotropic NMDA1 receptor. The DmGluRA antiserum is highly specific and has been used for analysis of both *Drosophila* neuromuscular junctions [60] and in the clock neuron circuits [85]. When applied to the *Drosophila* optic lobes distinct and strong immunolabeling was seen in the medulla and lobula complex, but not in the lamina (Figure S4C). In the lamina, the DmGluRA antiserum produced diffuse labeling that was hard to distinguish from background labeling. Several fixation protocols yielded the same result. The most likely site for glutamate release, the medulla terminals of L2, in particular, did not express presynaptic receptor immunolabeling.

The antiserum to the NMDA1 subunit was raised to a sequence of the protein that is quite well conserved between invertebrates and mammals, but has not been properly characterized on fly tissue. In a report on the lamina of another fly, *P. sericata*, the same antiserum was reported to label T1 processes in the lamina [47]. In spite of using the same protocol as these authors, and as well as testing several modifications (and different fixatives), we failed to obtain any proper immunolabeling in the lamina or medulla (Figure S4D1). We did, however, obtain strong immunolabeling with this NMDA1 antiserum in the mushroom body lobes (Figure S4D2), indicating that the antiserum recognized a *Drosophila* epitope. Possibly the lack of immunolabeling in the optic lobe reflected levels of receptor expression in *Drosophila* that were too low, or an inconvenient species difference.

### GABA signaling components in the lamina

#### GABA and GAD immunolabeling

**Figure 8:**
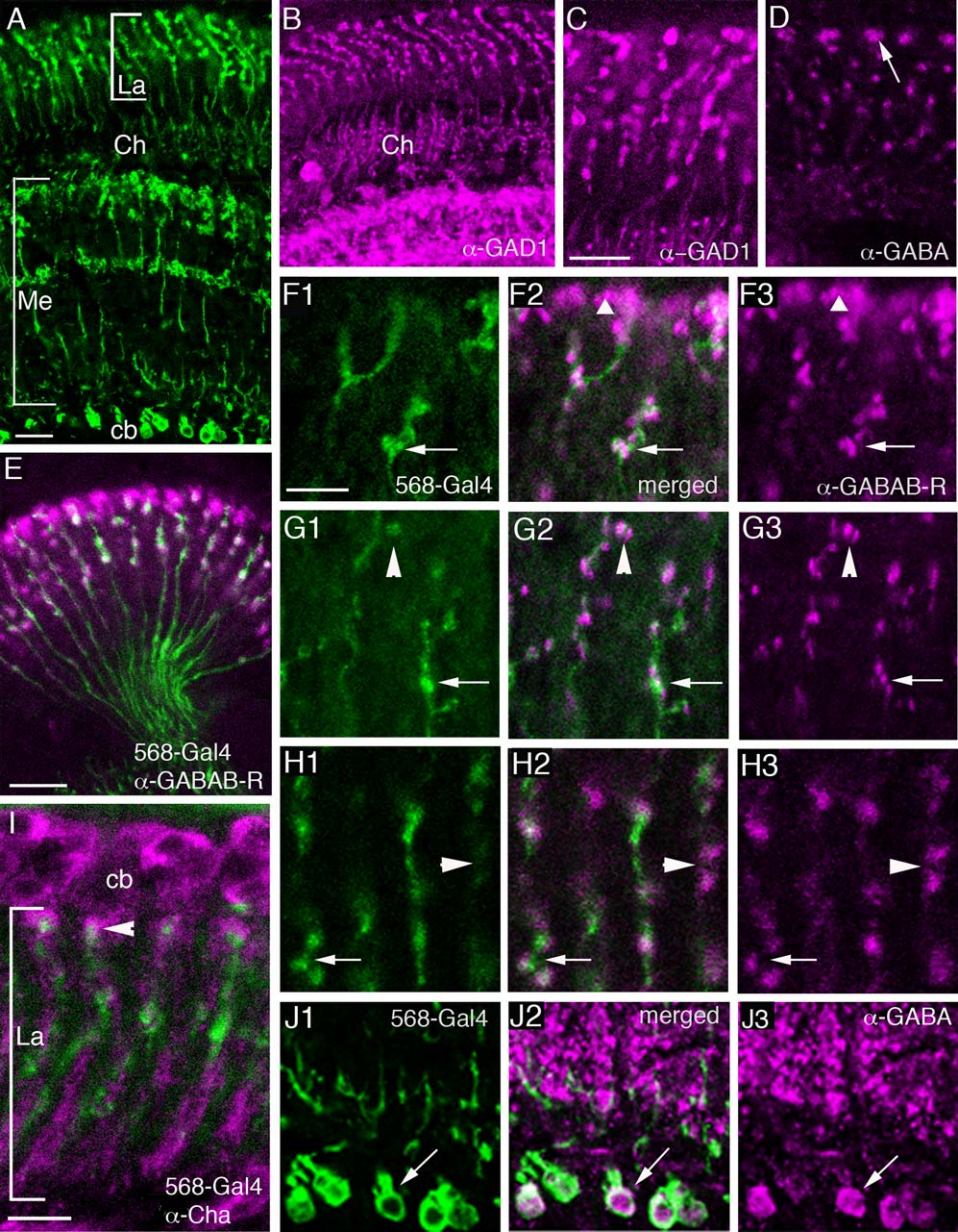
Centrifugal neurons in the lamina and markers for GABA signaling. A GFP expression in columnar C3 neuron terminals in the lamina (La) and medulla (Me) driven by the 5-6-8-Gal4. The C3 cell bodies (cb) proximal to the medulla are also visible. Ch: optic chiasma. Scale bar = 10 µm. B Distribution of glutamic acid decarboxylase-1 (GAD) immunoreactivity in C2 and C3 neurons (overview of lamina and part of medulla). Scale as in A. C Higher magnification of C2 and C3 terminals in lamina revealed by anti-GAD antiserum; note C3 varicosities along the axons. Scale bar = 10 µm. D Both C3 and C2 (labeled by arrow) terminals can be visualised in the lamina by anti-GABA antiserum. Same magnification as in C. E GFP in C3 neurons (in green) driven by 5-6-8-Gal4 with anti-GABA_B_R2 immunolabeling (magenta) in horizontal section of lamina. The C3 axons traverse the optic chiasma. Note that much of the receptor immunolabeling is in neurons other than C3, but that some appears co-localized (white). Scale bar = 10 µm. F–H Details of double-labeling with GFP expression in C3 neurons and antiserum to GABA_B_R2. Some GABA_B_ receptor expression is in C3 neurons (arrows in F, G and H). At other sites the receptor is expressed on neuronal structures that appear to be closely adjacent to C3 neurons (long arrowheads in G and H) or in C2 terminals indicated by short arrowhead in F. Scale bar for F–H (F1): 5 = µm. I C3 neurons (5-6-8-Gal4 driven GFP, green) do not co-localize ChAT immunoreactivity (magenta) but appear to be located close to ChAT immunolabeled profiles. Scale bar = 5 µm. J1–3 GFP-labeled C3 cell bodies (J1, green) express GABA-immunoreactivity (J3, magenta) as seen in merged image (J2). Scale as in F.

GABA is a major inhibitory neurotransmitter in *Drosophila* and other insects and distributed in large numbers of neurons [68], [86], [87]. Proven markers for GABAergic neurons are antisera to GABA, vesicular GABA transporter (vGAT) and the biosynthetic enzyme GAD. Here we employed GABA, vGAT and GAD (GAD-1) antisera to label lamina neurons. To identify C3 neurons we employed the 5-6-8-Gal4 line (Fig. 8A). Previous studies have shown that the centrifugal neurons C2 and C3 in different fly species display GABA immunoreactivity [42], [43], [44], [45]. Our study confirmed GABA and GAD immunoreactivity in C2 and C3 neurons in *Drosophila* (Fig. 8B–D, J). In a recent report from our laboratory [68] vGAT immunolabeling was also detected in C2 and C3 neurons. This suggests that the C2 and C3 neurons indeed both contain and utilize GABA as a neurotransmitter in the lamina: C2 probably releasing the transmitter from presynaptic sites that localized to enlarged boutons in a distal layer and C3 from similar sites at varicosities along their length in the lamina [9].

We analyzed the relations between the GFP-labeled C3 neurons (5-6-8-Gal4) and ChAT-immunolabeling and found no co-localization of markers (Fig. 8I). However, the C3 neurons were seen close to the ChAT-immunolabeled monopolar axons (which are most likely L4 neurons) and terminated close to the enlarged boutons of *Cha*-expressing tangential neurons, *Cha*-Tan.

#### GABA receptors

**Figure 9:**
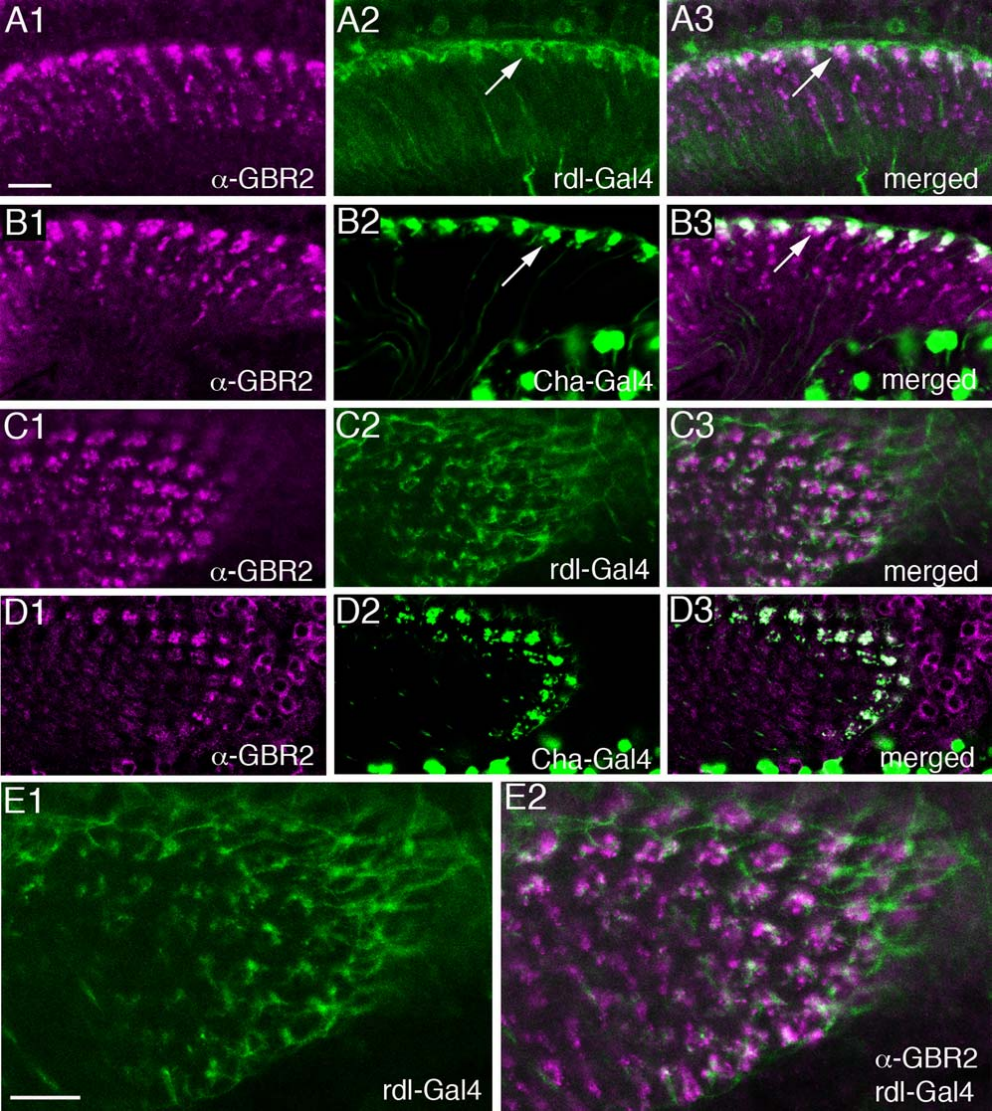
Comparison between *rdl*-Tan and *Cha*-Tan neurons in distal part of lamina. Arrows indicate differences in structures between the two types of tangential neurons. *rdl*-Tan have thin varicose processes hanging down into the lamina, whereas the *Cha*-Tan have enlarged boutons in the same layer. A (1–3) *rdl*-Tan neurons (A2, green), visualised by GFP expression driven by *rdl*-Gal4, contact GABA_B_R-immunolabeled cells at arrows, but do not coexpress the receptor (A1, magenta). B (1–3) *Cha*-Tan cells visualised by GFP expression driven by *Cha*-Gal4 (B2, green) co-express GABA_B_R immunoreactivity (B1, magenta) in their boutons (arrow). C (1–3) *rdl*-Tan neurons in cross-section are organized in widely branched network with arborizations in each cartridge (C2, green). Close contacts with GABA_B_R-immunopositive cells (C1, magenta) are visible. D (1–3) *Cha*-Tan neurons also are organized in a network but they have more distinct aggregates of boutons in each cartridge and these boutons co-express GABA_B_R-immunolabeling (schown in Fig. 8E) and co-localize anti-ChAT (D1, magenta, D3 – merged). Scale bar for images A to D = 10 µm. E (1–2) Higher magnification of the *rdl*-Tan processes distally in the lamina in cross section (E1) and contacts with GABA_B_R-immunolabeled neurons (E2). Scale bar = 10 µm.

The localization of the metabotropic GABA_B_ receptor 2 (GABA_B_R2) has previously been demonstrated in the brain of *Drosophila* by means of a specific antiserum [65], [68]. Here we show the distribution of GABA_B_R2 immunoreactivity (GBR-IR) in relation to the different lamina neurons visualized by GFP driven by specific Gal4-lines (Fig. 8E–H). The major expression of GBR-IR was seen on the distal varicosities of C2 neurons (Fig. 8F–H) and in boutons of C3 neurons (Fig. 8E–H), as well as on enlarged boutons of *Cha*-Gal4-expressing tangential neurons, *Cha*-Tan (Fig. 6E). GBR-IR expression in the lamina is thus likely to be localized presynaptically in C2 and C3 boutons and postsynaptically on the tangential neuron boutons. This would explain why the distribution of GBR-IR signal in this region appears in coherent aggregates larger than the C2 terminals and larger than the *Cha*-Gal4-expressing boutons (Fig. 6E). To investigate the relationship between C2 neurons and *Cha*-expressing neurons further, we also double-labeled tissues with anti-GABA_B_R2 antibodies and anti-ChAT (Fig. 9D). Again we saw that the immunolabeled *Cha*-Tan neuron boutons co-expressed GBR-IR material.

The GBR-IR material associated with the C3 neurons appear to be predominantly co-localized within the membranes of the C3 boutons throughout the depth of the lamina (Fig. 8F, G). This we interpret to represent presynaptic GABA_B_R2 in GABAergic C3s. However, some immunoreactivity seemed to lie in structures very close to these boutons, not co-localized to them (Fig. 8G, H). Thus, there is a possibility that a neuron postsynaptic to C3 also expresses GABA_B_R2 at sites located close to the contacts with C3 neurons. Given the restricted distribution of the immunoreactivity however we could not identify this neuron type. From EM analysis, possible candidates postsynaptic to C3 would be L1–L3 and amacrine cell processes [9], [13].

Ionotropic GABA_A_ type receptors are the major receptors mediating fast inhibitory transmission in the insect brain (see [87]). We tried to analyze the distribution of this type of receptor in the *Drosophila* visual system, but an antiserum to one of the *Drosophila* GABA_A_ receptor subunits, RDL, failed to produce distinct immunolabeling in the lamina, even though the medulla displayed layers of strong RDL-immunoreactivity (Figure S4E). An earlier report indicated diffuse immunolabeling possibly associated in part with large monopolar neuron dendrites [68]. Given this uncertainty, we resorted to using an *rdl*-promotor-Gal4 to drive GFP expression in neurons and reveal lamina expression of possible GABA_A_ receptors.

In the fly visual system, *rdl*-Gal4 expression (amplified by anti-GFP immunolabeling) was seen in two types of neurons in the lamina (Fig. 5B, 6B, 9A). It appears that one type is the L4 monopolar neurons. This is based on the small cell bodies just above the lamina neuropil and the short collateral branches in the proximal layer (Fig. 5B,D). The second type is a wide-field tangential neuron morphologically similar to the one designated La wf2 by Fischbach and Dittrich [12] (Figs. 5B, 6B, and 9B). We choose to refer to the latter neurons as *rdl*-expressing tangential (*rdl*-Tan) neurons. Since the RDL-antiserum does not yield strong immunolabeling we cannot confirm the Gal4 expression pattern in the lamina as representing RDL protein expression. However, in other parts of the *Drosophila* brain this Gal4 line seems to produce GFP expression that matches RDL-immunolabeling quite well (Enell and Nässel, unpublished). In double-labeled specimens, we detected no apparent co-localization of GABA_B_R2 immunolabeling and *rdl*-Gal4 expression in the distal portion of the lamina. Rather we noted a close association between the boutons of the *rdl*-Tan neurons and GBR-IR signal in C2 varicosities and/or large boutons of *Cha*-Tan neurons (Fig. 9A,C). If the *rdl*-Gal4 does in fact represent RDL distribution, there must be a differential distribution of GABA_A_ and GABA_B_ receptors in lamina neurons visualized in our study.

Another important finding was obtained by labeling with GABA signaling markers. The two Gal4 expression patterns in tangential processes seen in the distal lamina with *Cha*-Gal4 and *rdl*-Gal4 lines could be clearly distinguished. Using antiserum to GABA_B_R2 on *rdl*- and *Cha*-Gal4 flies it is clear that the *rdl*-Tan neurons are distinct from the tangential processes of the *Cha*-Tan neurons (Fig. 9A–E). Whereas the *Cha*-Gal4 expressing boutons co-express GABA_B_R2, the *rdl*-Gal4 ones do not. The boutons of these two types of tangential neurons are, however, in close proximity suggesting that they could both receive input from the same GABAergic C2 neurons. Confirmation of this possibility must await EM examination.

## Discussion

By combining immunocytochemistry with Gal4-directed GFP expression, we have mapped some components of the acetylcholine, glutamate and GABA signaling pathways in the peripheral visual system underlying the compound eyes of *Drosophila* (summarized in Table 2). We confirmed some previous reports for *Drosophila*: for example, the presence of GABA in the centrifugal neurons C2 and C3 [45] and the cholinergic phenotype of some lamina monopolar neurons [49], [51]. As discussed below, data to support a neurotransmitter function for glutamate in monopolar neurons L1–L2 are less decisive. We also have some new findings such as evidence for expression of choline acetyltransferase (ChAT) and vesicular acetylcholine transporter (vAChT) protein in the monopolar neuron L4, and expression of ChAT-immunolabeling and *Cha*-Gal4 driven GFP in what appears to be a previously unreported wide-field lamina tangential neuron, which we designate *Cha*-Tan. ChAT expression in *Drosophila* had previously been reported for somata of lamina monopolar cells in general [49], [51] and in *Calliphora* for amacrine neurons [88]. Another new finding is the presence of vesicular glutamate transporter (vGluT) immunoreactivity in what are probably the α-processes of lamina amacrine neurons. This finding confirms with a more reliable phenotypic marker earlier indications of glutamate immunoreactivity in amacrine cells of another fly species [47], which we extend with observations made here from electron microscopical immunocytochemistry. Attempts to map the distribution of GABA_A_ and GABA_B_ receptors, as well as ionotropic and metabotropic glutamate receptors in lamina circuits met with variable success. Only GABA_B_ receptors were clearly identifiable by immunocytochemistry in the lamina, although GABA_A_ receptors were expressed in the medulla. However, *rdl*-Gal4 driven GFP indicated possible expression of the GABA_A_ receptor subunit RDL in a wide-field tangential neuron (*rdl*-Tan), similar to a variant type of tangential neurons previously designated La wf2 [12], and in L4 monopolar neurons.

**Table 2:** Distribution of signaling components in fly lamina indicated by various markers.

Neuron type	Marker^1^	Tentative marker^2^	Reference^3^	This study	
				Receptor	Marker^4^
**R1–R6**	α-Histamine		8,9		-
**R7/R8**	α-Histamine	α-GABA /GAD	8,9, *3*		-
**L1**	α-Glutamate	α-ChAT, *cha* in situ	11, *12*		α-ChAT 5, α-vGluT^6^, α-Glutamate
**L2**	α-Glutamate α-RDL	α-ChAT, *cha* in situ	6,11, *12*		α-ChAT 5, α-vGluT^6^ α-Glutamate, vGluT-Gal4
**L3**	α-Glutamate		11		not detectable in *Drosophila*
**L4**	-				α-ChAT, α-vAChT Rdl-Gal4
**L5**	-				-
**C2**	α-GABA, α-GAD α-vGAT	α-ChAT	3,4,6,7,10, *2*	α-GABA_B_R	α-GABA, α-vGAT
**C3**	α-GABA, α-vGAT		10,11	α-GABA_B_R	GABA, GAD α-vGAT
**T1**	α-“NMDA-R1”		11		-
**Am**	α-Glutamate α-ChAT		11, *5*		α-vGluT 7
**Tan 1 ** 8 ***Cha*** **-Tan**				α-GABA_B_R	α-ChAT, *cha*-Gal4
**Tan 2** 8 ***rdl*** **-Tan**				*rdl*-Gal4	*rdl*-Gal4

Notes.

1 Immunocytochemical identification of putative neurotransmitter/substance, protein, biosynthetic enzyme or receptor in a specific neuron type. Evidence is more complete for underlined neuron types.

2 Tentative identification of neuron type with marker (no clear statement/commitment was made in papers).

3 References (the references listed to the right in column, in *italics*, refer to the tentative identifications): **1.** Barber et al. [51], **2.** Buchner et al. [89], **3.** Buchner et al. [44] **4.** Datum et al. [42], **5.** Datum et al. [88], **6.** Enell et al. [68], **7.** Meyer et al. [43], **8.** Nässel et al. [38], **9.** Sarthy [39], **10.** Sinakevitch et al. [45]. **11.** Sinakevitch and Strausfeld [47]. **12.** Yasuyama and Salvaterra [49].

4 Including Gal4 driven GFP.

5 Only cell bodies labeled with ChAT antiserum.

6 The vGluT immunolabeling seen only in L1 and L2 cell bodies, not processes.

7 The immunolabeling pattern resembles α- and or β-processes in lamina and since we detected no immunolabeled axons in the chiasma between lamina and medulla (but occational cell bodies in position of Am neurons), we assign the immunolabeling to α-processes of amacrine (Am) neurons.

8 The tangentially arranged processes detected with these markers do not completely match tangential neurons (La wf1) or amacrine neurons described from Golgi impregnations [12]. Thus we refer to them as *Cha*-Tan and *rdl*-Tan neurons. The *rdl*-Tan resemble the La wf2 neurons, a possible variant of La wf1 neurons [12].

### Acetylcholine signaling components

The best evidence for neurons qualified to use acetylcholine for signaling was obtained for the wide-field *Cha*-Tan neurons. Both *Cha*-Gal4 expression and the ChAT- and vAChT- antisera identify these neurons. The *Cha*-Tan neurons give rise to enlarged boutons, most probably associated with distal C2 neuron terminals. Thus, we believe they were mistaken for C2 neurons in earlier reports on ChAT-immunolabeling in flies [89]. Especially with Gal4-driven GFP expression it is clear that these large boutons are parts of the *Cha*-Tan neurons, and may thus be partly regions receiving input from centrifugal neurons. The *Cha*-Tan neurons also produce varicose processes that run between the boutons and that have short branches hanging down into the lamina synaptic neuropil. It therefore seems that a portion of the cholinergic neurotransmission from *Cha*-Tan neurons is confined to a shallow layer in the distal lamina. The wide spread of these processes was the reason that the synaptic connections of wide-field tangential neurons were not investigated by Meinertzhagen and O'Neil [9], so the synaptic targets or inputs of these neurons are still unknown in *Drosophila*. Another layer of cholinergic neurotransmission may occur in the proximal portion of the lamina, by means of collaterals of the L4 monopolar neurons.

Published reports on the immunocytochemical localization of acetylcholine receptors in the CNS of *Drosophila* have also shed some light on the lamina circuitry. Two nicotinic receptor subunits and the muscarinic receptor have previously been detected in the lamina [90], [91], [92]. Whereas the muscarinic receptor [92] and the ALS subunit were only seen weakly and diffusely distributed in this neuropil, the ARD subunit was revealed distally in the lamina in bouton-like clusters [90]. Thus, the ARD distribution closely matches that of the boutons of the *cha*-Tan neurons, but it is not clear what neuron type(s) expresses the receptor.

L4 monopolar neurons have three collateral processes in the basal portion of the lamina [12]. These interconnect the L4 neurons in adjacent cartridges, as well as the L2 cell and photoreceptor terminals within the neighboring and native cartridges, and appear to be the major outputs from the L4s within the lamina [9]. At intermediate pattern contrasts, L2 in *Drosophila* recruits L4 as the substrate for detection of front-to-back motion [93]. We find that the collateral branches of L4s strongly express ChAT and vAChT immunoreactivities, suggesting that a cholinergic pathway may be responsible for this recruitment in the lamina. In two earlier reports on ChAT-immunoreactivity in *Drosophila* [49], [89] the L4 collaterals are visible in the figures, but did not receive specific comment. Interestingly the *rdl*-Gal4 drives GFP in what appears to be L4 neurons (and a set of tangential neurons, *rdl*-Tan). The failure of our antiserum to provide matching immunocytochemical evidence for RDL expression in these neurons, means that it is not clear whether the neurons express this GABA_A_ receptor subunit, or – more likely – whether they may merely do so at levels too low to detect immunocytochemically. Overall, it is tempting to speculate that acetylcholine is used for lateral connections over larger or smaller areas of the lamina mosaic (respectively: *Cha*-Tan distally and L4 proximally).

As reported previously (see [49]), and confirmed in our study, the cell bodies of the large monopolar neurons L1 and L2 also express ChAT-immunoreactivity, and *Cha*-transcript [51] although we could not detect vAChT immunoreactivity in these neurons. Thus, it is not clear whether the large monopolar neurons utilize acetylcholine as a neurotransmitter, even though they may have a capacity to synthesize it, or whether the vesicular transporter is expressed at too low levels to detect.

### Glutamate signaling components

We validated previously published data, including our own [46] on glutamate signaling components by using different antisera to the *Drosophila* vesicular glutamate transporter (vGluT), as well as analyzing *vGluT*-Gal4 expression. Glutamate signaling seems to be performed at two main candidate sites in the lamina, large monopolar cells and amacrine neurons.

We obtained clear evidence for glutamate-like immunoreactivity in the large monopolar cells L1–L3 in the lamina and medulla of two fly species (*Musca* and *Calliphora*), whereas only two of these neurons, L1 and L2, were detected in *Drosophila*. There was, however, some variation in the latter species, L2 alone being invariably labeled. It most plausible to attribute this variation to different levels of cytoplasmic glutamate that could have existed under different functional states prior to preparation for immunolabeling. Compatible with these neurons having the capacity to store vesicular glutamate, the OK371-Gal4 line, specific for *vglut* expression, also drives GFP in the large monopolar neurons, but we did not detect clear vGluT immunolabeling in monopolar neurons. However, low levels of vGluT immunolabeling were seen in cell bodies of the large monopolar neurons and immunolabeling in dendrites of these neurons may be masked by the stronger immunolabeling seen in amacrine processes. Another more likely possibility is that the amount of vesicle-bound glutamate (and vGluT) is simply too low to detect. We thus do not have conclusive evidence that L1 and L2 have the capacity to store vesicular glutamate, and consequently that they are glutamatergic in *Drosophila*. These neurons have most of their synaptic output in the medulla and either no (L1) or a limited number (L2) of output synapses in the lamina [9], [13]. Insofar as L1 sometimes clearly expresses a glutamate phenotype but lacks output synapses in the lamina, we would predict the absence of glutamate containing vesicles and corresponding vGluT in the lamina, at least in L1. On the other hand, as revealed by the 21D-Gal4 line, we did not detect vGluT immunolabeling in the L2 medulla terminals either, again possibly because there was insufficient protein to yield a clear immuno signal. Thus it still cannot be entirely excluded that the glutamate immunoreactivity seen previously [46], [47] may represent non-vesicular amino acid stored as a metabolic intermediate. On the other hand, the possibility that these monopolar neurons, the major output neurons of the fly's lamina, might use two fast neurotransmitters, glutamate and acetylcholine, may not be unprecedented [94]. However, our evidence provides no support for the possibility that the cells might release these at different sites, or even in different neuropils (L1 in the medulla, and L2 also in the lamina).

While this paper was in revision an elegant study appeared on the distribution of a vesicular glutamate transporter in *Drosophila* [95]. The authors of that report used a different *vglut* promoter Gal4, but one of the vGluT antisera [61] also used in our investigation. Although the paper did not report on vGluT distribution in the lamina, the authors report expression in the medulla, where, as in our study, they found no conclusive evidence for *vglut* or vGlut expression in the terminals of L1 to L3.

In addition to the monopolar neurons, strong vGluT immunolabeling was seen in structures resembling the α-processes of amacrine neurons, and this could be correlated with immunoreactivity to glutamate seen by electron microscopical analysis. Sinakevitch and Strausfeld [47] also detected such immunoreactivity in the fly *Phaenicia sericata*, thus providing some measure of support for a glutamatergic phenotype in the lamina amacrine cells.

Overall, there are some incongruencies in the data for glutamate signaling: the processes of monopolar neurons express immunoreactivity for glutamate but not the vesicular transporter, while the amacrine cells express immunolabeling for the transporter but not the expected Gal4 expression. To resolve some of these issues, we had hoped to see DmGluRA expression in lamina circuits, but very weak labeling was seen and this could not readily be assigned to any specific neuron type. Since we detected very strong DmGluRA immunolabeling in neurons of the medulla, we presume the expression level is just very low in the lamina neurons. It was therefore surprising that the antiserum to the NMDA1 receptor subunit used in a previous study [47] labeled neither neither in the lamina nor elsewhere in the visual system. The antiserum was raised against a sequence of a mammalian NMDA1 receptor protein with limited similarities to that in *Drosophila* and thus not likely to display much cross-reactivity in *Drosophila*. However, we could show rather strong immunolabeling of neurons in the mushroom body lobes, suggesting that again the lack of signal could be a matter of low levels of expression in the visual system of *Drosophila*.

Adopting cautious criteria, we can summarize the positive findings on glutamate signaling components in the lamina as follows. We find evidence that the α-processes of lamina amacrine neurons express vGluT, and glutamate. These neurons, which we might therefore predict to be glutamatergic, have many outputs onto β-profiles of T1 neurons, and onto R1–R6 and L1–L3 neurons [9]. Compatible with this suggestion, Sinakevitch and Strausfeld [47] reported NMDA1 receptor-like immunoreactivity on T1 neurons in *P. sericata*. Glutamate may thus be used as a transmitter in amacrine neurons for wide-field interconnections (see also [29], [30]). We also entertain the possibility that lamina monopolar neuron L2 may use glutamate for signaling within the lamina at some of its many minority classes of synapses, but that neither L1 nor L2 shows clear evidence of doing so at their chief output terminals in the medulla.

### GABA signaling

Our immunocytochemical data show that C2 and C3 neurons (identified by Gal4-driven GFP) express both GABA and GAD. Neither of these neurons was detected using a GAD1-Gal4 line [96] tested here, and no other lamina neuron clearly expressed GAD1 or GABA immunoreactivity. An exclusive GABA phenotype among centrifugal neurons is confirms earlier reports on *Drosophila* and other flies [42], [43], [44], [45]. Previously we have also shown that the C2 and C3 neurons express the *Drosophila* vesicular GABA transporter [68], further suggesting that these neurons signal by means of GABA.

We localized GABA_B_R immunoreactivity in relation to various identified neurons. For this we used an antiserum to the GABA_B_R2, a G-protein coupled receptor known to dimerize with the GABA_B_R1, to form a functional receptor complex [97], [98]. Thus our observations are likely to reveal functional GABA_B_ receptor sites (see [68]). At least three neuron types seem to express GABA_B_Rs: C2, C3, and the tangential neuron *Cha*-Tan. Possibly there is an additional neuron type not identified that may be postsynaptic to the C3 neurons that express GABA_B_Rs, since we also see immunoreactivity adjacent to C3's boutons. The likely contacts between GABAergic C2 neurons and large boutons of *Cha*-Tan neurons are quite distinct and express high levels of GABA_B_ receptor immunoreactivity. The presence of GABA_B_R on C2 terminals in the distal lamina indicates the presence of presynaptic GABA receptors at a GABA output site of these neurons. Similarly GABA_B_R immunoreactivity is associated with the varicosities of the GABAergic C3 neurons. These varicosities can be assumed to be GABA release sites, and are known to provide input to L1–L3 and amacrine cell processes and to receive no inputs themselves [9], [13]. Thus the GABA_B_ receptor may be presynaptic in both the C2 and C3 neurons. Both pre- and postsynaptic locations of GABA_B_Rs have in fact been identified in mammals (see [99]). There, presynaptic GABA_B_R activation inhibits transmitter release by inhibiting voltage-gated Ca^2+^ channels via the β/γ subunit of the G-protein, or by inhibiting adenylate cyclase via G_i/o_ proteins [99]. In this way, GABA release from C2 or C3 may be negatively regulated.

The distribution of GABA_A_ type receptors in the lamina is still not clear, because the antiserum to the *Drosophila* GABA_A_ receptor subunit RDL failed to produce distinct lamina immunolabeling. An earlier study suggested that at least part of the RDL-immunolabeling may be localized to L2 monopolar cells [68]. Here we utilized an *rdl*-Gal4 line to drive GFP, and although we were unable to demonstrate that lamina neurons revealed by *rdl*-Gal4 actually produce RDL, a good match between the markers has been seen in many parts of the larval CNS (Enell and Nässel, unpublished). The *rdl*-Gal4 labels L4 monopolar neurons and *rdl*-Tan neurons. At least *rdl*-Tan neurons may be targets of GABAergic C2 neurons as seen in our study, whereas the L4 neurons are not known to be postsynaptic to either C2 or C3 neurons [9], so that receptor expression on these monopolar cells is unexplained and may be targeted to the medulla terminals.

In summary, GABA seems to be primarily (or exclusively) used by centrifugal neurons from the medulla with outputs in the lamina, one of which (C2) may signal to wide-field tangential neurons of the lamina.

### Conclusions

This study has increased the number of lamina neurons for which a putative neurotransmitter has now been identified and has also localized GABA_B_ receptors to identified neurons (see Table 2). There are, of course, still many neuron types for which transmitters remain unknown. Perhaps the greatest mystery of all remains whether the large monopolar neurons utilize glutamate or acetylcholine as neurotransmitters, or whether they may possibly release both. They appear qualified to use either, but it is neither clear which they actually use, nor whether release is the same at sites in the lamina and medulla, or in different strata of these neuropils. It is also evident that glutamate receptors and RDL subunits of GABA_A_ receptors are expressed at levels too low to be reliably detected in the lamina. Our study now prompts the complete morphological characterization of the possibly novel types of tangential neurons *Cha*-Tan and *rdl*-Tan. These are perhaps variants of the La wf1 and 2 neurons already described. It is also urgent to determine the neurotransmitter of the L1 and L2 neurons and to localize ionotropic receptors for acetylcholine, GABA and glutamate in the lamina circuits.

Since, in contrast to the lamina of locusts, cockroaches or other non-dipteran insects [100], [101], [102], it appears that lamina interneurons in flies express neither monoamines such as histamine, dopamine, octopamine or serotonin (see [35], [103]) nor identified neuropeptides, further work will be required to screen for small-molecule neurotransmitters in those neurons not yet assigned a signal molecule. Co-expression of yet unidentified neuromodulators clearly remains an additional possibility, revealed for example by dense-core vesicles in C2 ([9]: their Fig. 36A), alongside the clear vesicles which we may now presume to contain GABA. Thus, the complete neurotransmitter repertoire of even the tiny constituency of neurons in the fly's lamina cartridge still awaits final identification.

## Supporting Information

Figure S1 Confocal examination of glutamate-like immunoreactivity in the optic lobes of Musca and Calliphora. A–D: Musca. A: Tangential section of the lamina, revealing the array of cartridges, and the repeated pattern of immunoreactive profiles. B: Horizontal section, showing longitudinally sectioned axon profiles in the lamina, and medulla, and the heavy labeling in the external chiasma between the two neuropils. C: At higher magnification, each cartridge is revealed by large immunoreactive profiles at its core (small circle) circumscribed by a ring of small profiles (within the large circle) contributed by α-processes of amacrine cells. The perikarya of some monopolar cell somata in the lamina cortex also exhibit faint immunoreactivity. D: Paired axon profiles (circles) are especially clear deep in the proximal lamina, in a section plane that cuts the adjacent chiasma. E: Calliphora. Tangential section of the medulla reveals not only immunoreactive chiasmal fibers, as seen in Musca (B,D) but also their axon profiles and terminals in the array of medulla columns, and tangential fibers. Scale bar: 1 µm.iles and terminals in the array of medulla columns, and tangential fibres. Scale bar: 1 µm.
(8.83 MB TIF)

Figure S2 Immuno-EM labeling of lamina cartridges in Musca is localized to L1–L3. A: Immuno-labeled profiles in three cartridges exhibit darkened cytoplasm and microtubules in L1 and L2 (L) and illustrate the excellent state of ultrastructural preservation of surrounding elements of the cartridge. Immunosignal stops at the base of a labeled axon, probably L1 (arrow). Scale bar: 1.0 µm. B The profiles of monopolar cells L1–L3 are immunolabeled, L2 possibly more darkly. The axons of the long visual fibres (7,8) are also faintly immunoreactive. Scale bar: 1.0 µm. C Profiles of a single cartridge. Surrounding photoreceptor terminals are well preserved, with clear profiles of tetrad synapses (arrowheads), in one case at a site providing input upon L2 (arrowhead). Scale bar: 1.0 µm.
(5.19 MB TIF)

Figure S3 Immuno-EM labeling of lamina cartridges at proximal depths in the lamina (Musca). A Example of L2 feeding synaptic input back upon surrounding R1–R6 terminals, in this case at the unusually large number of three profiles of such sites (arrowheads) on two terminals in this single section. Scale bar: 0.5 µm. B Single lamina cartridge revealing immuno-labeled profiles of L1–L3. L2 has the profile of a single presynaptic site (arrowhead). Scale bar: 1.0 µm. C Enlarged L2 feedback synaptic profile shown in B, upon the profile (asterisk) of what probably derives from the basket endings of the medulla cell T1, the normal postsynaptic partner to a receptor terminal profile, such as that from the nearby R1. Note increased density of the presynaptic ribbon, and of the surrounding synaptic vesicles. Scale bar: 0.25 µm. D Profile of L2 feedback synapse, similar to that in C, but in which the receptor terminal (R) and likely T1 profile (asterisk) share the postsynaptic locations. Scale bar: 0.25 µm.
(4.90 MB DOC)

Figure S4 Distribution of vGluT immunolabeling in processes of amacrine neurons in lamina of Drosophila. Strong immunolabeling is seen in α-processes in the lamina and weaker label in cell bodies in the chiasma (arrow). Scale bar = 10 µm. B1 Cross section of the lamina reveals topology of vesicular glutamate transporter (vGluT) immunoreactive processes (in magenta) in relation to photoreceptors labellabeled with antiserum to discs large, DLG (green). The vGluT expression is seen in processes in positions like α-processes of amacrine cells (or β-processes of T1 neurons). B2 Anti-vGluT labeling in relation to monopolar neurons revealed by OK371-Gal4 driven GFP (green). B3 Anti-vGluT labeling in relation to monopolar cells revealed by 21D-Gal4. Scale bar = 5 µm. C1 Metabotropic glutamate receptor A (DmGluRA) immunolabeling is seen in medulla layers (Me) but not in lamina neuropil (La). Scale bar = 20 µm. C2 Higher magnification and increased imaging intensity reveals strong labeling in medulla neurons (including cell bodies), but very weak and diffuse labeling in the lamina. Scale bar = 10 µm. D (1–2) NMDAR1 immunolabellabeling in optic lobe is weak and diffuse (D1) whereas in the central brain (D2) it was possible to detect NMDAR1 expression in the mushroom body lobes (MB). Scale bar = 10 µm. E Distribution of RDL-immunolabeling in medulla and lobula (Lo). In the lamina only weak and diffuse labeling was seen (not shown). Scale bar = 20 µm.
(4.78 MB TIF)

Figure S5 Attempts to correlate the distribution of vesicular glutamate transporter (vGluT) immunolabeling with structures revealead by Gal4-driven GFP. Here we used an n-synaptobrevin-GFP fusion (nsyb-egfp) to direct GFP mainly to synaptic terminals (green). A The 21D-Gal4 drives nsybGFP primarily in the medulla (Me) terminals of the L2 neurons. Commensurate with the 10-fold fewer presynaptic sites in the lamina (13), than the medulla [1], almost no GFP is visible in the lamina (La). B Details of nsyb-eGFP expressing L2 terminals in the medulla in oblique cross section. C The same terminals seen with vGluT immunolabeling. The two labels do not co-localize, indicating that the L2 neurons do not express vGluT in the medulla. D1–D3 Frontal sections of the medulla showing L2 terminals displayed by 21D-Gal4 crossed to UAS-nsyb-eGFP and vGluT immunolabeling (magenta). Again there is no co-localization of labels. E1–E3 Frontal sections of medulla comparing the distribution of OK371-Gal4-driven nsyb-eGFP and vGluT immunolabeling. In contrast to many other parts of the brain the two markers do not co-localize in most structures, except partly in the inner medulla layers (IL). In particular, clear-cut labeling was seen in neither the L1 nor L2 terminals. Reference 1. Takemura S, Lu Z, Meinerzhagen IA (2008) Synaptic circuits of the Drosophila optic lobe: the input terminals to the medulla. J Comp Neurol (in press)
(12.08 MB TIF)
