# Supplementary figures and images for: Glutamate, GABA and Acetylcholine Signaling Components in the Lamina of the Drosophila Visual System
Source: PLoS One. 2008 May 7;3(5):e2110. doi: 10.1371/journal.pone.0002110 (PMC2373871; doi:10.1371/journal.pone.0002110)

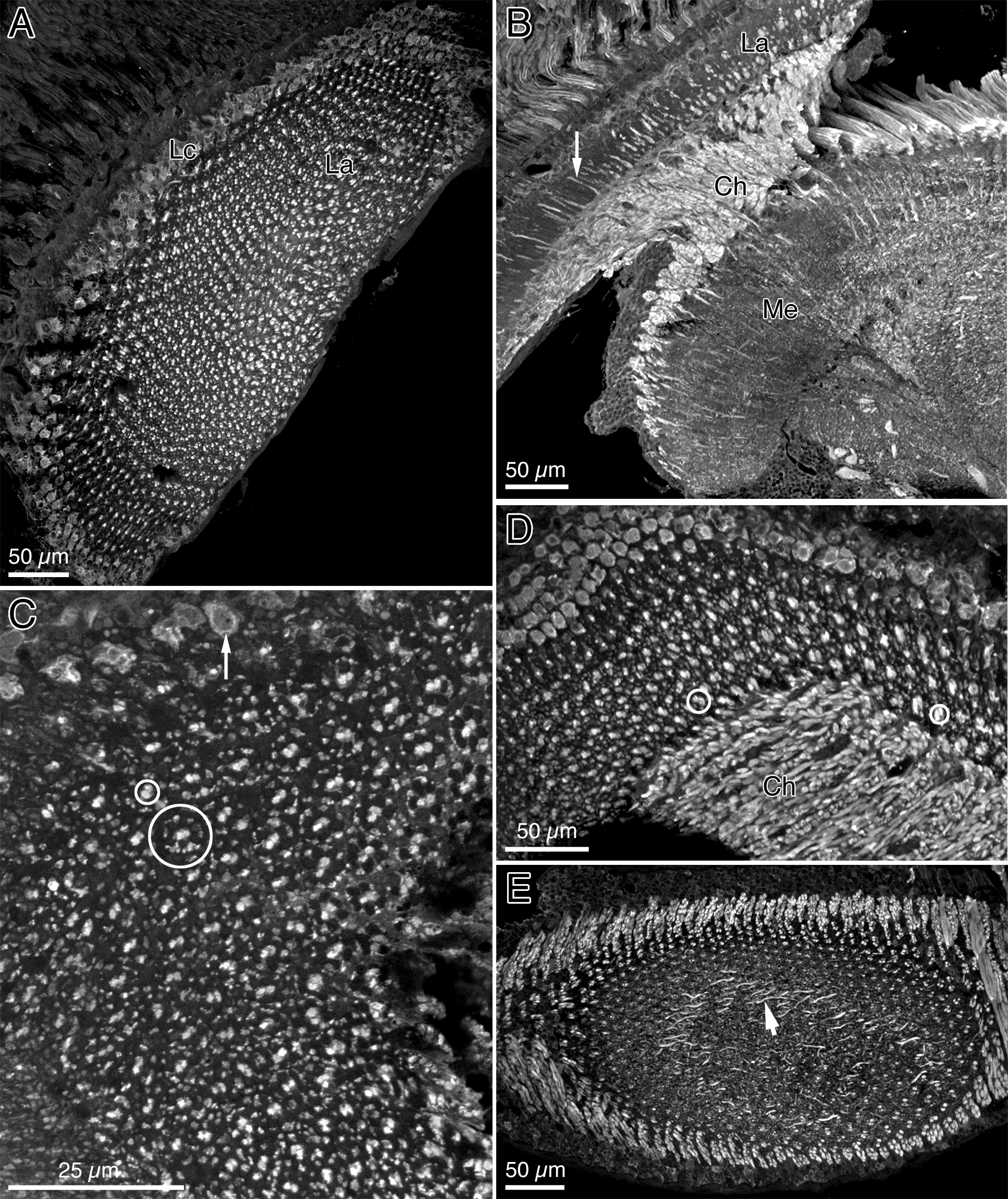

Supplement: Figure S1 — Confocal examination of glutamate-like immunoreactivity in the optic lobes of Musca and Calliphora. A–D: Musca. A: Tangential section of the lamina, revealing the array of cartridges, and the repeated pattern of immunoreactive profiles. B: Horizontal section, showing longitudinally sectioned axon profiles in the lamina, and medulla, and the heavy labeling in the external chiasma between the two neuropils. C: At higher magnification, each cartridge is revealed by large immunoreactive profiles at its core (small circle) circumscribed by a ring of small profiles (within the large circle) contributed by α-processes of amacrine cells. The perikarya of some monopolar cell somata in the lamina cortex also exhibit faint immunoreactivity. D: Paired axon profiles (circles) are especially clear deep in the proximal lamina, in a section plane that cuts the adjacent chiasma. E: Calliphora. Tangential section of the medulla reveals not only immunoreactive chiasmal fibers, as seen in Musca (B,D) but also their axon profiles and terminals in the array of medulla columns, and tangential fibers. Scale bar: 1 µm.iles and terminals in the array of medulla columns, and tangential fibres. Scale bar: 1 µm. (8.83 MB TIF) [file pone.0002110.s001.tif]

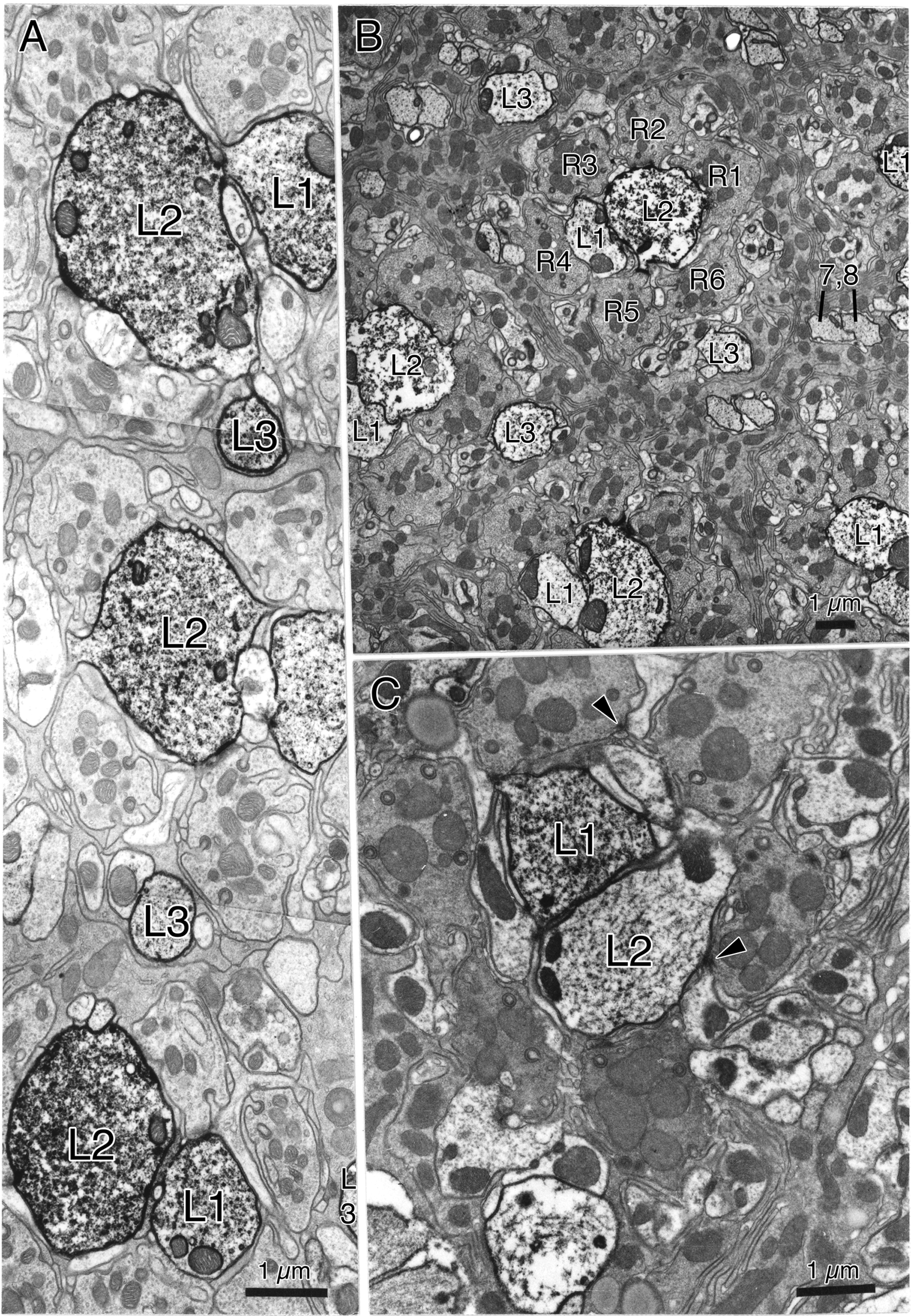

Supplement: Figure S2 — Immuno-EM labeling of lamina cartridges in Musca is localized to L1–L3. A: Immuno-labeled profiles in three cartridges exhibit darkened cytoplasm and microtubules in L1 and L2 (L) and illustrate the excellent state of ultrastructural preservation of surrounding elements of the cartridge. Immunosignal stops at the base of a labeled axon, probably L1 (arrow). Scale bar: 1.0 µm. B The profiles of monopolar cells L1–L3 are immunolabeled, L2 possibly more darkly. The axons of the long visual fibres (7,8) are also faintly immunoreactive. Scale bar: 1.0 µm. C Profiles of a single cartridge. Surrounding photoreceptor terminals are well preserved, with clear profiles of tetrad synapses (arrowheads), in one case at a site providing input upon L2 (arrowhead). Scale bar: 1.0 µm. (5.19 MB TIF) [file pone.0002110.s002.tif]

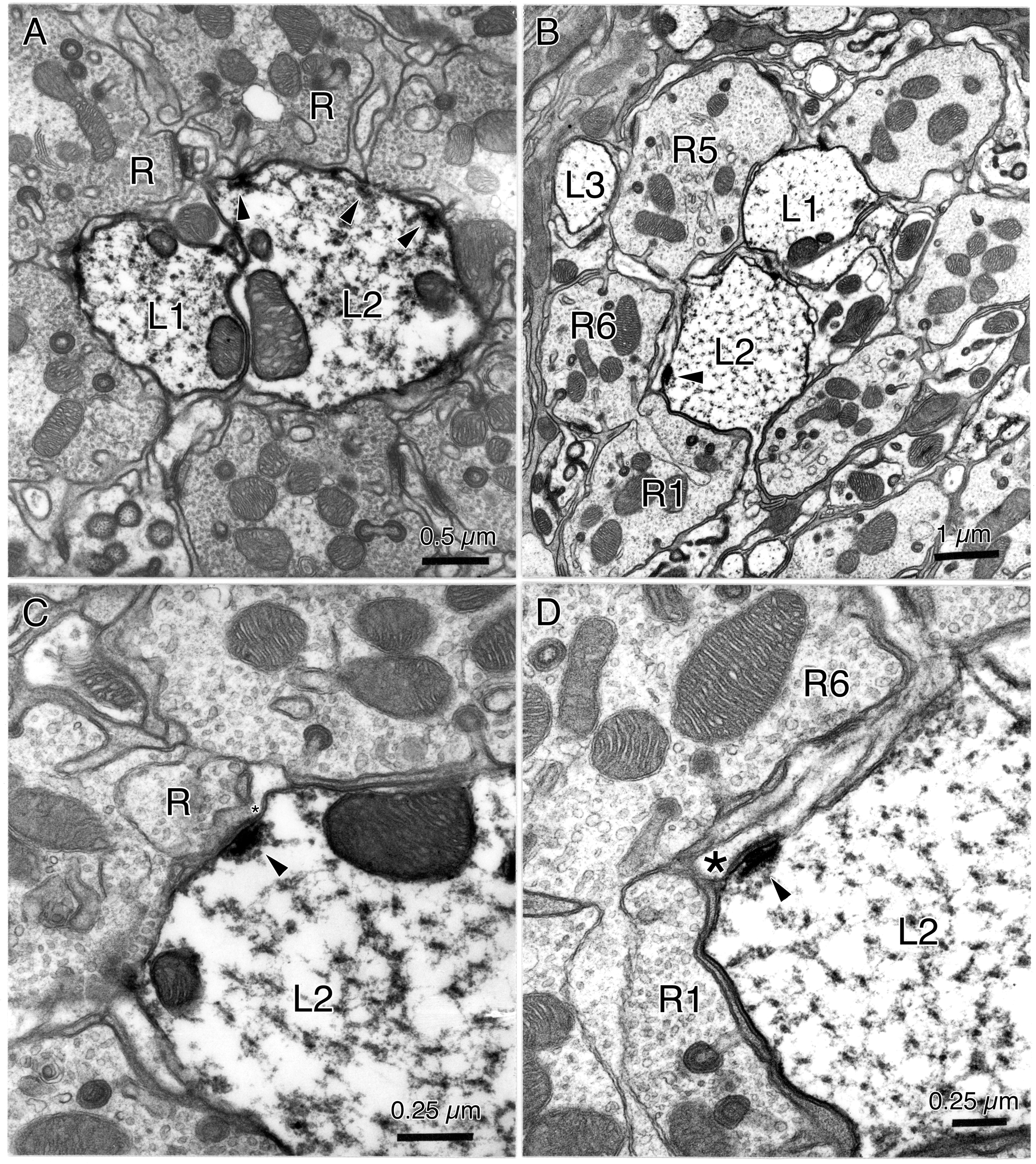

Supplement: Figure S3 — Immuno-EM labeling of lamina cartridges at proximal depths in the lamina (Musca). A Example of L2 feeding synaptic input back upon surrounding R1–R6 terminals, in this case at the unusually large number of three profiles of such sites (arrowheads) on two terminals in this single section. Scale bar: 0.5 µm. B Single lamina cartridge revealing immuno-labeled profiles of L1–L3. L2 has the profile of a single presynaptic site (arrowhead). Scale bar: 1.0 µm. C Enlarged L2 feedback synaptic profile shown in B, upon the profile (asterisk) of what probably derives from the basket endings of the medulla cell T1, the normal postsynaptic partner to a receptor terminal profile, such as that from the nearby R1. Note increased density of the presynaptic ribbon, and of the surrounding synaptic vesicles. Scale bar: 0.25 µm. D Profile of L2 feedback synapse, similar to that in C, but in which the receptor terminal (R) and likely T1 profile (asterisk) share the postsynaptic locations. Scale bar: 0.25 µm. (4.90 MB DOC) [file pone.0002110.s003.tif]

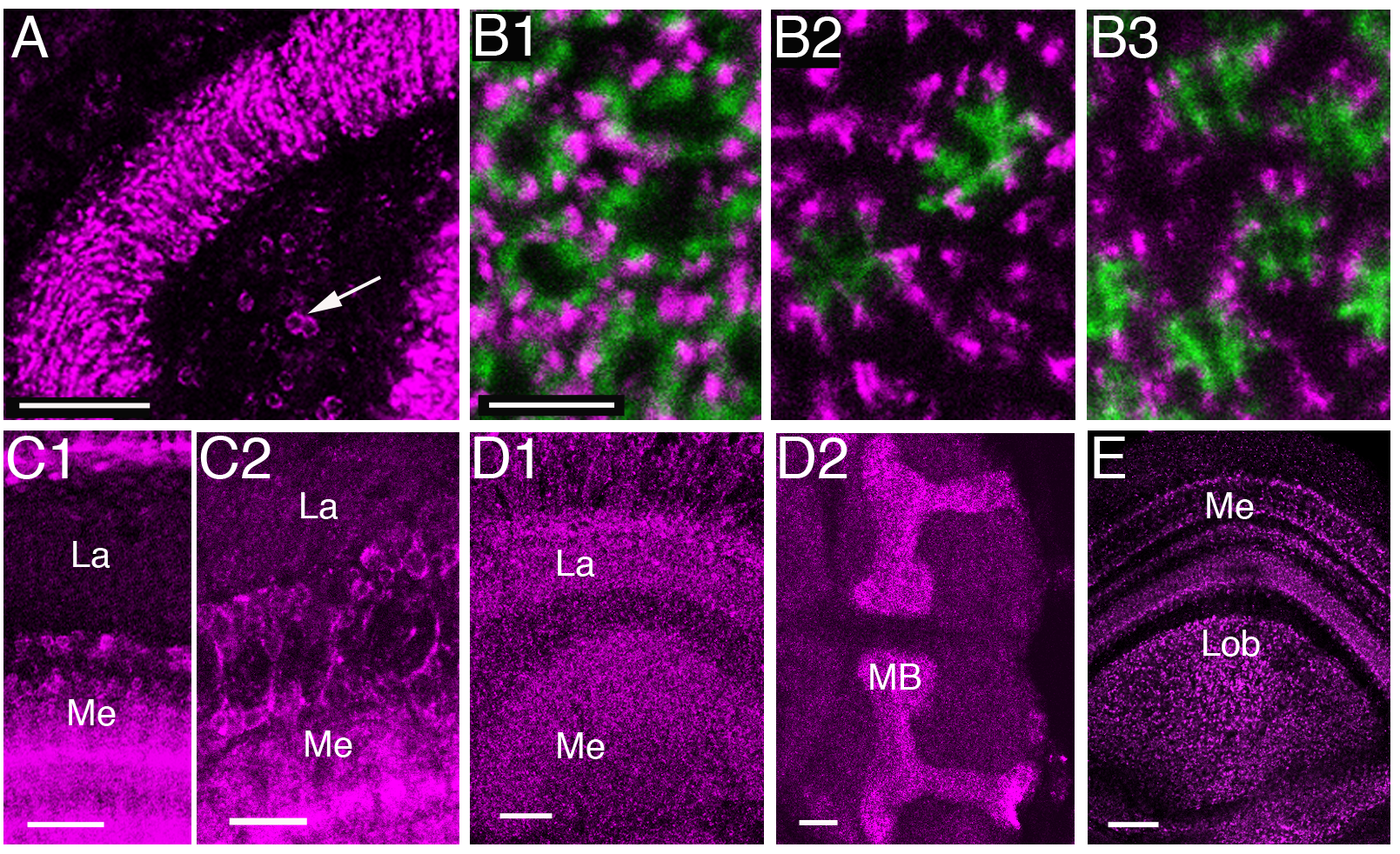

Supplement: Figure S4 — Distribution of vGluT immunolabeling in processes of amacrine neurons in lamina of Drosophila. Strong immunolabeling is seen in α-processes in the lamina and weaker label in cell bodies in the chiasma (arrow). Scale bar = 10 µm. B1 Cross section of the lamina reveals topology of vesicular glutamate transporter (vGluT) immunoreactive processes (in magenta) in relation to photoreceptors labellabeled with antiserum to discs large, DLG (green). The vGluT expression is seen in processes in positions like α-processes of amacrine cells (or β-processes of T1 neurons). B2 Anti-vGluT labeling in relation to monopolar neurons revealed by OK371-Gal4 driven GFP (green). B3 Anti-vGluT labeling in relation to monopolar cells revealed by 21D-Gal4. Scale bar = 5 µm. C1 Metabotropic glutamate receptor A (DmGluRA) immunolabeling is seen in medulla layers (Me) but not in lamina neuropil (La). Scale bar = 20 µm. C2 Higher magnification and increased imaging intensity reveals strong labeling in medulla neurons (including cell bodies), but very weak and diffuse labeling in the lamina. Scale bar = 10 µm. D (1–2) NMDAR1 immunolabellabeling in optic lobe is weak and diffuse (D1) whereas in the central brain (D2) it was possible to detect NMDAR1 expression in the mushroom body lobes (MB). Scale bar = 10 µm. E Distribution of RDL-immunolabeling in medulla and lobula (Lo). In the lamina only weak and diffuse labeling was seen (not shown). Scale bar = 20 µm. (4.78 MB TIF) [file pone.0002110.s004.tif]

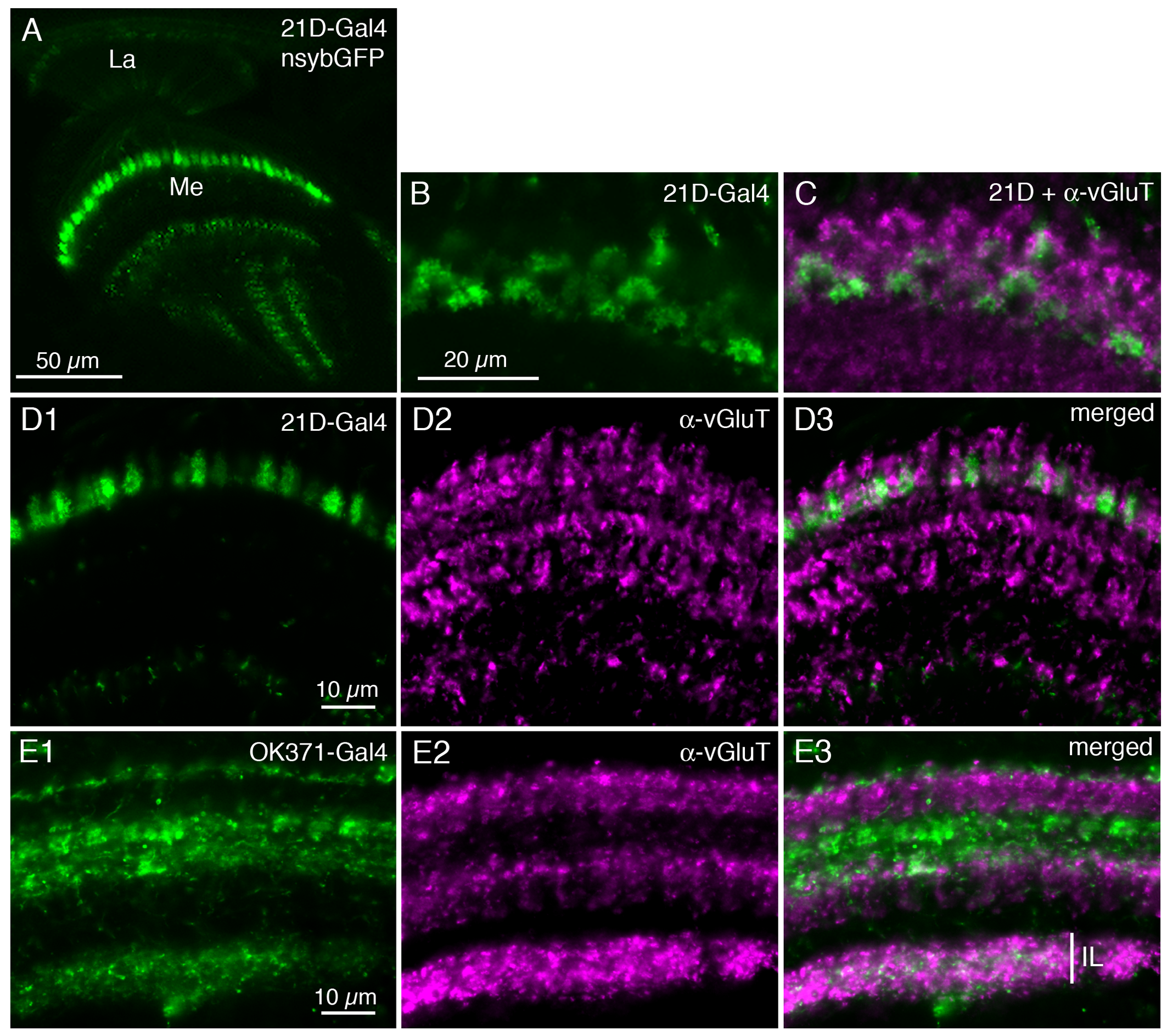

Supplement: Figure S5 — Attempts to correlate the distribution of vesicular glutamate transporter (vGluT) immunolabeling with structures revealead by Gal4-driven GFP. Here we used an n-synaptobrevin-GFP fusion (nsyb-egfp) to direct GFP mainly to synaptic terminals (green). A The 21D-Gal4 drives nsybGFP primarily in the medulla (Me) terminals of the L2 neurons. Commensurate with the 10-fold fewer presynaptic sites in the lamina (13), than the medulla [1], almost no GFP is visible in the lamina (La). B Details of nsyb-eGFP expressing L2 terminals in the medulla in oblique cross section. C The same terminals seen with vGluT immunolabeling. The two labels do not co-localize, indicating that the L2 neurons do not express vGluT in the medulla. D1–D3 Frontal sections of the medulla showing L2 terminals displayed by 21D-Gal4 crossed to UAS-nsyb-eGFP and vGluT immunolabeling (magenta). Again there is no co-localization of labels. E1–E3 Frontal sections of medulla comparing the distribution of OK371-Gal4-driven nsyb-eGFP and vGluT immunolabeling. In contrast to many other parts of the brain the two markers do not co-localize in most structures, except partly in the inner medulla layers (IL). In particular, clear-cut labeling was seen in neither the L1 nor L2 terminals. Reference 1. Takemura S, Lu Z, Meinerzhagen IA (2008) Synaptic circuits of the Drosophila optic lobe: the input terminals to the medulla. J Comp Neurol (in press) (12.08 MB TIF) [file pone.0002110.s005.tif]
